# Treatment With FoxP3+ Antigen-Experienced T Regulatory Cells Arrests Progressive Retinal Damage in a Spontaneous Model of Uveitis

**DOI:** 10.3389/fimmu.2020.02071

**Published:** 2020-09-04

**Authors:** Yi-Hsia Liu, Christine Mölzer, Kimmo Makinen, Koju Kamoi, Clare L. C. Corbett, Izabela P. Klaska, Delyth M. Reid, Heather M. Wilson, Lucia Kuffová, Richard J. Cornall, John V. Forrester

**Affiliations:** ^1^Institute of Medical Sciences, University of Aberdeen, Aberdeen, United Kingdom; ^2^MRC Human Immunology Unit, MRC Weatherall Institute of Molecular Medicine, University of Oxford, Oxford, United Kingdom

**Keywords:** autoimmune uveitis, cell therapy, adoptive transfer, hen-egg lysozyme, T cell anergy, anti-uveitogenic

## Abstract

We specify the clinical features of a spontaneous experimental autoimmune uveitis (EAU) model, in which foreign hen-egg lysozyme (HEL) is expressed in the retina, controlled by the promoter for interphotoreceptor retinol binding protein (IRBP). We previously reported 100% P21 (post-partum day) IRBP:HEL single transgenic (sTg) mice, when crossed to transgenic T cell receptor mice (3A9) generating the double transgenic (dTg) genotype, develop EAU despite profound lymphopenia (thymic HEL-specific T cell deletion). In this work, we characterized the immune component of this model and found conventional dTg CD4+ T cells were less anergic than those from 3A9 controls. Furthermore, prior *in vitro* HEL-activation of 3A9 anergic T cells (T_an_) rendered them uveitogenic upon adoptive transfer (Tx) to sTg mice, while antigen-experienced (AgX, dTg), but not naïve (3A9) T cells halted disease in P21 dTg mice. Flow cytometric analysis of the AgX cells elucidated the underlying pathology: FoxP3+CD25^hi^CD4+ T regulatory cells (T_reg_) comprised ∼18%, while FR4+CD73+FoxP3-CD25^lo/–^CD4+ T_an_ comprised ∼1.2% of total cells. Further T_reg_-enrichment (∼80%) of the AgX population indicated FoxP3+CD25^hi^CD4+ T_reg_ played a key role in EAU-suppression while FoxP3-CD25^lo/–^CD4+ T cells did not. Here we present the novel concept of dual immunological tolerance where spontaneous EAU is due to escape from anergy with consequent failure of T_reg_ induction and subsequent imbalance in the [T_reg_:T_effector_] cell ratio. The reduced numbers of T_an_, normally sustaining T_reg_ to prevent autoimmunity, are the trigger for disease, while immune homeostasis can be restored by supplementation with AgX, but not naïve, antigen-specific T_reg_.

## Introduction

The relative contribution of T cell anergy (T_an_) vs. regulatory T cells (T_reg_) in the context of autoimmunity has become blurred as a result of recent studies in evaluating the relationship between T_reg_ and T_an_ (1). This has particular relevance to the development of customized cell therapies for immune-mediated diseases, some of which are close to clinical translation. Standardized protocols for preparing T regulatory cells (T_reg_) and tolerogenic dendritic cells (tolDC) have been proposed (2, 3) and are aimed at facilitating progress toward Phase III clinical trials for autoimmune and immune-mediated diseases (4) but limited account is taken of the role of T_an_ in these protocols (4). Uveitis is an immune-mediated/autoimmune disease of considerable morbidity (4–6) in which the inciting agent is obscure (7). Current treatment options are limited to disease control by steroids and biologics. Experimental models of autoimmune uveoretinitis (EAU) have been helpful in exploring disease mechanisms (8). Traditionally, EAU is induced by subcutaneous inoculation of retina-specific antigens (9) in complete Freund’s adjuvant (CFA). However, such conventional models may not be representative of “non-infectious” human uveitis, which develops in the absence of an obvious trigger, i.e., spontaneously (7).

Critically, experimental models of spontaneous uveitis, mostly in genetically manipulated mice represent an important attempt to mimic clinical disease more closely. In some models, single transgenic (sTg) mice express “foreign” antigens such as β-galactosidase or hen egg lysozyme (HEL) in the retina or lens under the control of a tissue-specific promoter such as rhodopsin, α-crystallin, arrestin, or interphotoreceptor retinol binding protein (IRBP). Uveitis can either be induced after adoptive transfer (Tx) of antigen-specific T cells (10) or develops spontaneously in F1 mice after crossing sTg mice to antigen-specific T cell receptor (TCR) mice, thus generating the double-transgenic (dTg) HEL/TCR genotype (11). In the IRBP:HEL sTg mouse model, HEL protein is photoreceptor membrane-bound, linked to the MHC class I protein. In dTg mice EAU develops with a 100% frequency (11), while 3A9 TCR (HEL-specific) mice do not develop uveitis. Both rhodopsin-HEL and IRBP:HEL sTg mice develop a degree of retinal photoreceptor shortening without inflammation, which, in the case of IRBP:HEL sTg mice is associated with reduced levels of IRBP. This retinal degeneration is age-related and has no impact on the development of EAU in dTg mice which has its onset at P21 when the retina is normal and there is abundant retinal HEL expression (12).

In the present study, we report that dTg mice undergoing extensive thymic clonal deletion of HEL-specific CD4+ T cells with profound lymphopenia evident from P20/21 (11), develop clinical signs of EAU at the same time with focal patches of retinal vasculitis and infiltration of the retina by Th1 and Th17 cells. Spontaneous development of EAU in dTg mice correlates with both, lymphopenia affecting particularly the T_reg_ compartment, and limited T cell anergy. Adoptive transfer of HEL-specific (1G12+) CD3+CD4+CD8- conventional T cells (T_conv_) to young (P21) sTg mice also causes EAU in a dose-dependent manner. Pathogenic T_conv_ cells secrete both IFNγ and IL17/IL22 and require activation by HEL *in vitro* to induce EAU on Tx. HEL-specific (1G12+) CD3+CD4-CD8- double negative (DN) cells are also present in dTg mice but are not pathogenic.

Interestingly, Tx of unfractionated antigen-experienced (AgX, P60) lymph node T cells from dTg mice with end-stage EAU, but not lymph node (LN) cells from 3A9 TCR mice, arrested the development of EAU and even reversed disease. FoxP3+CD25+ T_reg_ were found to be the suppressive T cell population. These data attest to an imbalance between T_reg_ and T_eff_ that permits spontaneously activated, poorly anergic T_conv_ to induce disease (13).

The mechanisms in pre-clinical models of EAU are under continued investigation. Here, we characterized in detail the immune component driving the pathogenesis of our spontaneous model of EAU. We furthermore show that (a) both limited anergy and an imbalance in [T_reg_:T_eff_] combine to permit development of spontaneous autoimmunity; that (b) treatment with AgX T_reg_ can prevent spontaneous autoimmunity; and that (c) protocols to generate T_reg_ *in vitro* may need to take into account the proportion of T_an_ in the cell preparation.

## Materials and Methods

### Study Design

Transgenic IRBP:HEL mice were used to investigate in detail the clinical dynamics and severity of spontaneous autoimmune uveitis (EAU) in the dTg genotype, using *in vivo* and *in vitro* methodological approaches, including the therapeutic adoptive transfer of an enriched T_reg_ cell population.

### Animals

The generation of dTg mice was previously described (11, 12). The procedures adopted conformed to the regulations of the Animal License Act (United Kingdom). All mice were bred in established breeding colonies and housed in a Medical Research Facility, University of Aberdeen. The genotype of the mice was verified by genotyping using standard in-house PCR procedures. Littermate male and female mice of different ages and genotypes were used in the experiments as specified, with 3A9 TCR mice serving as control animals.

### Clinical Evaluation of Ocular Disease

Mice fundi were imaged using an otoscope-based fiber-optic light device as described previously (14). Following the Laboratory Animal Science Association’s (LASA) good practice guidelines for administration of substances, mice were anaesthetized with an intraperitoneal injection of a mixture of 40 mg/kg Vetalar^®^ (Fort Dodge Animal Health Ltd., Southampton, United Kingdom) and 0.2 mg/kg Domitor^®^ (Orion Pharma, Espoo, Finland) diluted in injectable water. Pupils were dilated with Minims^®^ 1% (w/v) Tropicamide, and 2.5% (w/v) Phenylephrine hydrochloride (both from Bausch & Lomb UK Ltd., Kingston-upon-Thames, United Kingdom). Viscotears^®^ Carbomer 2 mg/g liquid gel (Alcon Eyecare UK Ltd., Camberley, United Kingdom) was applied to the corneal surface to protect the cornea from drying during imaging. Severity of disease was graded on the appearance and number of fundus lesions using an inflammation scoring system modified from Xu et al. (14) (Supplementary Table 1) and an atrophy scoring system (12) (Supplementary Table 2).

### Histology

Mice (P16-60) were sacrificed and eyes removed immediately. One eye was fixed in 2.5% (w/v) glutaraldehyde (Fisher Chemicals, Loughborough, United Kingdom) and embedded in resin for standard hematoxylin and eosin (H&E) staining. Images were collected using a ProgRes XT Core 5 color digital microscope camera (JENOPTIK Optical Systems GmbH, Jena, Germany) mounted on an inverted microscope (Carl Zeiss Axioskop 40, MicroImaging GmbH, Jena, Germany). The fellow eye was prepared for immunostaining.

### Immunostaining

Freshly collected eyes were embedded in Tissue-Tek^®^ OCT compound embedding medium (Sakura Finetek Europe B.V., Alphen aan den Rijn, Netherlands), and cryo-sections (7 μm) cut and fixed with cold acetone for 15 min at room temperature (20 ± 2°C). After 3 thorough washes, samples were blocked with 5% BSA for 30 min and were then incubated with the following antibodies (all purchased from BD Biosciences, Oxford, United Kingdom, unless stated otherwise): rabbit anti-HEL (Rockland Immunochemicals, Limerick, PA, United States; diluted 1:300 in PBS), anti-mouse CD4 FITC (GK1.5, 1:20), purified anti-mouse CD4 (clone GK1.5, 1:100), anti-mouse CD3 FITC (KT3, 1:20), anti-mouse MHCII FITC (28-16-8S, 1:20), biotinylated anti-mouse MHCII (28-16-8S, 1:20), anti-mouse CD11b FITC (M1/70, 1:100), purified anti-mouse CD11c (HL3, 1:50), purified anti-mouse CD8 (53-6.7, 1:100), anti-mouse F4/80 FITC (CI:A3-1, 1:20) for 1h, followed by TRITC conjugated anti-rabbit IgG and Cy5-conjucated streptavidin (anti-HEL), Alexa 546-conjugated anti-rat streptavidin (for CD4), Alexa 546-conjugated anti-rat streptavidin (for MHCII), Alexa 546-conjucated anti-hamster streptavidin, Cy5-conjugated anti-hamster streptavidin (for CD11c), Alexa 546-conjugated anti-rat streptavidin (for CD8) for a further hour. The dilution factor of the secondary antibodies was 1:200. After staining, samples were washed and mounted with Hydromount^TM^ Aqueous Non-fluorescing Mounting Media (National diagnostics, Hull, United Kingdom), and photos taken using a Zeiss LSM510 confocal microscope (Carl Zeiss Meditec, Göttingen, Germany).

### Flow Cytometry

Single cell suspensions of cells isolated from retinas and lymph nodes (15) were analyzed using flow cytometry. Both eye-draining (submandibular) and non-draining LN (superficial cervical and inguinal) were included. For analysis of eye-infiltrating cells, both retinal tissues were separated from the choroid. Retinas were digested for 40 min at 37°C in 1 ml PBS containing final conc. of 10 μg/ml Liberase and 10 μg/ml DNase I (both from Roche, Mannheim, Germany). Dissociated cells were washed and re-suspended in PBS containing 2% FBS for staining. Primary antibodies used (all from BD Biosciences, Oxford, United Kingdom) were as follows: *Fc*-receptors were blocked for 10 min (4°C) using CD16/32 (2.4G2) antibody. Cells were then surface stained with directly conjugated monoclonal antibodies including CD25 (PC61) PE and/or CD4 (GK1.5) APC-Cy7. In some experiments, 1G12 primary antibody to HEL-specific TCR (clone: 1G12; cell line kindly provided by Professor Goodnow, Australian National University) with secondary APC-conjugated anti mouse IgG1 (X56) was included. Alternatively, for a different batch of experiments, anti-Vβ8.1/8.2 TCR was used (BD BV605-conjugated; MR5-2; BD Biosciences, Oxford, United Kingdom). For analysis of T_reg_, cells were stained intracellularly using a FoxP3 staining kit (APC/eFluor^®^ 450 conjugated FoxP3, FJK16S) according to manufacturer instructions (eBioscience, Hatfield, United Kingdom). As an additional marker for T_reg_, folate receptor 4 antibody (anti-FR4 PerCp-Cy5.5-conjugated; 12A5, Biolegend, London, United Kingdom) was used. For detection of T cell anergy, anti-CD73 (AF700-conjugated; Ty11.8; Biolegend, London, United Kingdom) was chosen. The gating strategy for the phenotypic CD4+ T cell characterization is provided in Supplementary Figures 1a–f). For intracellular cytokine staining, dissociated cells were washed and re-suspended in culture media for further *ex vivo* stimulation. Cells were incubated for 5h in RPMI medium containing 10% FBS (both from Gibco, Fisher Scientific UK Ltd., Loughborough, United Kingdom), 50 ng/ml phorbol 12-myristate-13-acetate (16) and 1 μM ionomycin (both from Sigma-Aldrich, St. Louis, MO, United States) for 5h in the presence of monensin (BD GolgiStop^TM^, BD Biosciences, Oxford, United Kingdom). Next, *Fc*-receptors were blocked, cells were surface stained with anti-CD4 APC-Cy7 and 1G12 antibody followed by fixation using BD Cytofix/Cytoperm^TM^ (BD Biosciences, Oxford, United Kingdom). The following antibodies were used for intracellular staining: anti-mouse IFNγ (XMG1.2) with APC; IL17A (TC11-18H10) with PE-CF594; and IL22 (1H8PWSR) with PE. In all experiments, dead cells were excluded using a dead cell exclusion dye (Fixable Viability Dye eFluor 455, eBioscience, Hatfield, United Kingdom; or eFluor 506, Biolegend, London, United Kingdom). For all flow cytometry experiments 1–2 × 10^5^ events were acquired on a BD LSRII flow cytometer (BD Bioscience, Oxford, United Kingdom). Generated data were analyzed using FlowJo^®^, LLC for Windows, version 10 (TreeStar Inc., Ashland, OR, United States). Leukocytes were gated on a forward scatter (FSC-A) vs. side scatter (SSC-A) dot plot, followed by exclusion of cell aggregates using a forward scatter pulse gate (FSC-H vs. FSC-A). All subsequent analyses were based on live cells only, where unstained cells served as the gating control. Gates for individual markers of interest were set based on FMO- or isotype-controls, respectively, with acceptable background/unspecific staining signals of ≤1% of parent.

### Isolation of 1G12+DN Cells

1G12+DN cells were aseptically purified from lymphoid tissues of adult 3A9 TCR mice. Spleen and lymph nodes (submandibular, superficial cervical, axillary, and inguinal LN) were collected (separately or pooled) and mechanically disrupted using 40 μm nylon cell strainers. Single cell suspensions were incubated with CD4 (L3T4) MicroBeads and the CD4- cells collected, followed by a purification step. Briefly, a positive selection was performed, using magnetic bead-filled columns. Single cell suspensions from the above tissues were run through the column once, followed by 5 washes using wash buffer (3 ml each) [0.5% (v/v) bovine serum albumin (BSA), and 2 mM EDTA in Ca^2+^/Mg^2+^ free phosphate buffered saline (PBS) (all from Gibco, Fisher Scientific UK Ltd., Loughborough, United Kingdom)]. The eluted CD4- cells were collected and further depleted of potentially remaining CD4+ contaminating cells, using a CD4+ T cell Isolation Kit (MACS Miltenyi Biotec, Surrey, United Kingdom), following the manufacturer’s protocol. Using flow cytometry, the purity of the 1G12+DN cell population was assessed to be around 80%.

### Activation of Lymphocytes by HEL Protein and Anti-CD3/CD28 Antibodies

Activation of pooled lymph node lymphocytes from adult 3A9 mice, either with 1 μM HEL protein (Sigma-Aldrich, St. Louis, MO, United States) or with anti-CD3 (clone 17A2)/CD28 (clone 37.51) antibodies (both USB Molecular Biology Reagents: VWR International Ltd., Lutterworth, United Kingdom) was performed as described previously (17). The lymphocytes were washed and re-suspended at a density of 0.5 × 10^6^ cells/ml in complete medium [RPMI, 10% (v/v) FBS, 1% penicillin/streptomycin (v/v); (all from Gibco, Fisher Scientific UK Ltd., Loughborough, United Kingdom)] containing 200 pg/ml rIL-2 (Calbiochem Nottingham, United Kingdom), followed by addition of spleen feeder cells from B10. BR mice (5 × 10^4^ cells/well). Three days later, cells were harvested and re-suspended in PBS for further use.

### Adoptive Transfer

Transfer of activated, purified populations of CD4+ T_conv_, 1G12+CD3-CD4- (DN) T cells and unfractionated lymphocytes was prepared as outlined above and described previously (17). Cells were injected intravenously (*i.v.;* dorsal tail vein) into IRBP:HEL sTg mice of different age. EAU was clinically monitored by fundoscopy on day 5, day 8 and day 10 post-injection. After the last images had been taken on day 10, the animals were sacrificed, and eyes removed immediately for histological evaluation.

### T_reg_ Cell Isolation

T_reg_ cells were aseptically purified from 3A9 TCR and dTg HEL/TCR mice aged between P50 and P70. Spleen and LN (submandibular, superficial cervical, axillary, and inguinal) were collected and pooled single cell suspensions passed through a CD4+CD25+ regulatory T cell isolation kit of CD4 (L3T4) MicroBeads UK followed by magnetic microbead positive and negative selection according to the manufacturer’s manual. In some procedures, CD127+ (IL7R) T cells were removed by a further pass through a column containing CD127-antibody labeled beads as described (18). The percentage of FoxP3+, CD73+ and FR4+ T cells was evaluated in the purified populations by flow cytometry, and the CD4+ cells were found to be ∼80% T_reg_.

### T_reg_ Suppression Assay

To test T_reg_ suppression activity *in vitro*, equal numbers of CFSE labeled CD4+CD25- T_eff_ cells and mitomycin C (Sigma-Aldrich Company Ltd., Gillingham, Dorset, United Kingdom) treated antigen presenting cells (APC; 5 × 10^4^/well) were incubated for 3 days (5% CO_2_, 37°C) with 1 μM HEL protein (Sigma-Aldrich, St. Louis, MO, United States), and serial dilutions of purified T_reg_ ([T_reg_:T_eff_] ratios of [0.5:1] to [4:1]), followed by flow cytometric assessment of T_eff_ cell proliferation by evaluating progressive attenuation of CFSE staining as described previously (19).

### T Cell Anergy Assay

The ability of purified CD4+ cells from dTg and 3A9 mice, respectively, to proliferate *in vitro* was assessed following a previously published protocol (11). CD4+ cells were labeled with CFSE and challenged with serial dilutions of HEL protein (10^–5^–10^–11^ M) in the presence of mitomycin C-treated spleen APC for 3 days, after which cell proliferation was evaluated as stated above.

### Statistical Analysis

Statistical analysis was performed using GraphPad Prism for Windows, version 5 (La Jolla, CA, United States). Histograms were generated to check data distribution. For parametric data, one- or two-way ANOVA with Tukey’s Multiple Comparison *post hoc* Test for inhomogeneous variances, and Student’s *t-*test were used. The Mann-Whitney *u-*Test was applied on non-parametric data. Asterisks denote significant *p-*values based upon a 95% level of confidence (^∗^*p* < 0.05, ^∗∗^*p* < 0.01, ^∗∗∗^*p* < 0.001, ^****^p < 0.0001).

## Results

### EAU in dTg Mice Progresses From Patchy Vasculitis at P20 to Severe Retinitis at P44

**FIGURE 1:**
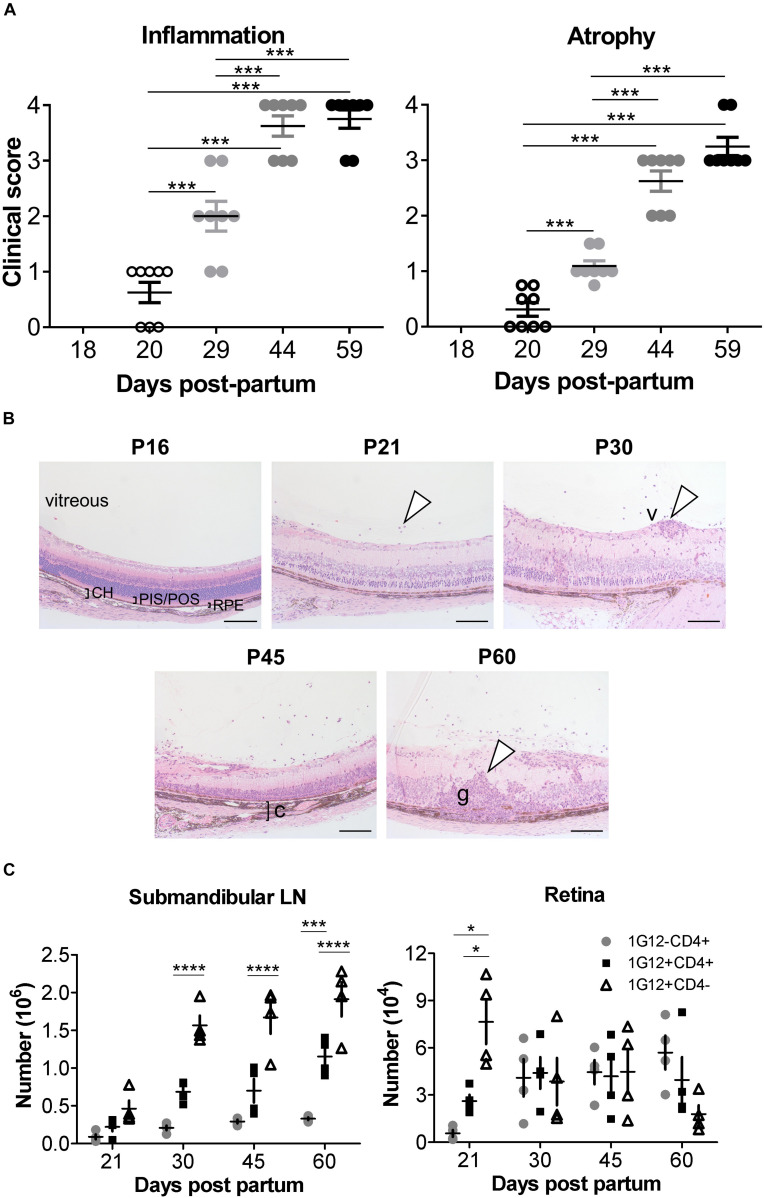
Clinical signs and phenotypic features of ocular inflammation in dTg HEL/TCR mice. **(A)** Retinal inflammation and atrophy were scored separately according to Supplementary Tables 1, 2, respectively, from post-partum day P20 to P59 (*n* = 8/group). Chronologically, atrophic changes followed the inflammatory signs. Data were analyzed using one-way ANOVA and Tukey’s Multiple Comparison *post hoc* Test with ***p < 0.001 when compared with P20 controls on a 95% level of confidence. **(B)** Eyes from dTg mice aged from P16 to P60 were fixed in 2.5% glutaraldehyde, embedded in resin, sectioned, and stained by hematoxylin and eosin (H&E). From P21, infiltrating cells were seen in the vitreous (arrowhead). From P30 to P60, vasculitis (v,arrowhead), choroiditis (c,bracket), and granuloma (g,arrowhead) were observed. CH, choroid; PIS/POS, photoreceptor inner-/outer layer; RPE, retinal pigment epithelium. Photos were taken using a ProgRes XT Core 5 color digital microscope camera (JENOPTIK Optical Systems GmbH, Jena, Germany) and mounted on a Zeiss Axioskop 40 microscope (Carl Zeiss, MicroImaging GmbH, Jena, GE). Scale bar: 100 μm. Representative images are shown. **(C)** Absolute number of cells found in the submandibular/eye-draining lymph nodes (LN) and the retina, respectively, during the course of EAU in dTg mice are shown (*n* = 4/age group). The numbers of 1G12+ double negative (DN), 1G12+CD4+ and non-antigen-specific CD4+ T cells increased steadily with age in dTg mice. The first cells populating the eye-draining LN and retinas of dTg mice were mostly 1G12+DN. The numbers of 1G12+DN cells decreased with age in the retinas of dTg mice, whereas non-antigen-specific (naïve) CD4+ increased in the later stage of EAU. Data were analyzed using one-way ANOVA and Tukey’s Multiple Comparison *post hoc* Test with *p < 0.05, ***p < 0.001, ****p < 0.0001 on a 95% level of confidence. Cell numbers are the average of 4 pairs of retina or DLN, i.e., cell count/pair. 1 × 10^5^ total events were recorded. Total numbers provided were extrapolated based on total cell counts, determined using a Coulter cell counter prior to sample processing.

We previously reported, using histological evaluation, the time of onset (P22) and incidence (100% at > P42) of spontaneous EAU in dTg mice (11). Here, using clinical fundoscopy to record global changes in the eye, we extend previous studies and more precisely time the onset of disease to P20 and fully characterize the progression of EAU in this model to peak severity of inflammation at P44 (Figure 1A, left panel and Supplementary Figure 2). In these more detailed studies, the dominant sign is inflammatory vasculitis followed by late extensive paravenous atrophy (Figure 1A, right panel and Supplementary Figure 2) and in ∼30% of cases eventual phthisis bulbi (globe shrinkage) (Supplementary Figure 2, gray box). Disease is absent in P18 dTg mice but presents clinically at P20 in around 60% of mice as focal patches of vasculitis (“cuffing”) of the major retinal vessels (Figure 1A and Supplementary Figure 2). By P29 all mice show signs of disease but with a range of clinical severity from small patches of retinal vasculitis to extensive vasculitis affecting all major retinal vessels and marked cellular infiltration of the vitreous gel (“vitreous haze”), obscuring retinal detail in some cases. This variable pattern continues with milder disease taking longer to reach grade 4 severity while more rapidly developing disease has begun to resolve by P44. This is accompanied by expanding areas of severe retinal atrophy particularly at sites of continuing severe vasculitis (Figure 1A and Supplementary Figure 2). By P59 active inflammation, including retinal and vitreous hemorrhage, persists in mice with slower onset disease while in more rapid onset disease, severe paravenous atrophy involving large parts of the retina has developed, with reduced inflammation mainly in the form of residual vitreous haze (Supplementary Figure 2). Retinal atrophy is accompanied with “pipe-stem” sheathing and severe “straightening” of the retinal vessels (Supplementary Figure 2, white arrowhead).

The two major manifestations of disease (inflammation and atrophy) were sufficiently different to merit a dual grading scheme (Supplementary Tables 1, 2). Atrophic retinal changes generally occurred at sites of resolving vasculitis accounting for the paravenous distribution, which spread to involve large areas of retina (Figure 1A and Supplementary Figure 2). The atrophic changes occurred with a slightly slower kinetic than the inflammatory disease (Figure 1A and Supplementary Figure 2).

In these experiments, we precisely pinpointed the clinical signs of EAU to progress from patchy vasculitis at P20, to severe retinitis at P44 (gradually leveling-off thereafter) and affecting dTg mice with a 100% incidence.

### Double-Transgenic HEL/TCR Mice Retain Lymphopenia Throughout Adulthood

The next experiments further defined the exact phenotype of our dTg mice focusing on the progression of their inherent T cell thymic clonal deletion over time. We previously reported, dTg mice are profoundly lymphopenic compared to 3A9 TCR mice (11), affecting particularly the CD4+ T cell compartment (Supplementary Figure 3 and Table 3). At P21 the percentage of CD4+ T cells is ∼4% in dTg mice compared to ∼25% in 3A9 mice. In time, there is gradual expansion in CD4+ populations, but in dTg mice lymphopenia persists to P60 (∼20% cells in dTg vs. ∼45% in 3A9 mice; end of observation) (Supplementary Figure 3). Despite the very low T cell numbers in P21 dTg mice, EAU onset occurred at this time as seen both clinically (Figure 1A and Supplementary Figure 2) and histologically (Figure 1B). Since EAU in dTg mice is due to CD4+ HEL-specific T cells (11), we asked what percentage of T cells in lymphopenic dTg mice were HEL-specific and how many HEL-specific T cells infiltrated the retina.

HEL-specific T cells express the Vβ8.2 TCR chain in the 3A9 TCR mouse and can be identified using the 1G12 monoclonal antibody (20). We found that the 1G12 antibody also detects a DN CD3+CD4-CD8- T cell population. In the eye-draining lymph nodes (DLN) of dTg mice at all stages of disease, the 1G12+DN population was consistently greater than the 1G12+CD4+ population. The 1G12+CD4+ population also increased with time but remained significantly lower than the total 1G12+CD4- T cell population (Figure 1C), as previously reported in other models (21, 22). In the retina, DN cells formed the greater part of the early T cell infiltrate at onset of disease (P21: ∼7.5 × 10^4^/retina) but declined steeply in number by P30 and were infrequent at P60 (Figure 1C). Conversely, 1G12+CD4+ T cells, were considerably less frequent at onset of disease (P21, ∼3.0 × 10^4^/retina) but increased and remained stable in absolute numbers to P60. Non-antigen-specific 1G12-CD4+ T cells also increased through P60 and became the most frequent cell type in the retina by that time (P60, ∼6 × 10^4^/retina) (Figure 1C). These data suggest selective recruitment/accumulation of antigen-specific CD4+ T cells over time. We hypothesize that the prominent infiltration of DN T cells identified at the onset of disease may facilitate later accumulation of CD4+ T cells by initiating breakdown of the blood-retinal barrier.

### Retinal Granulomas Are Abundant in dTg Mice and Resemble Tertiary Lymphoid Organs

**FIGURE 2:**
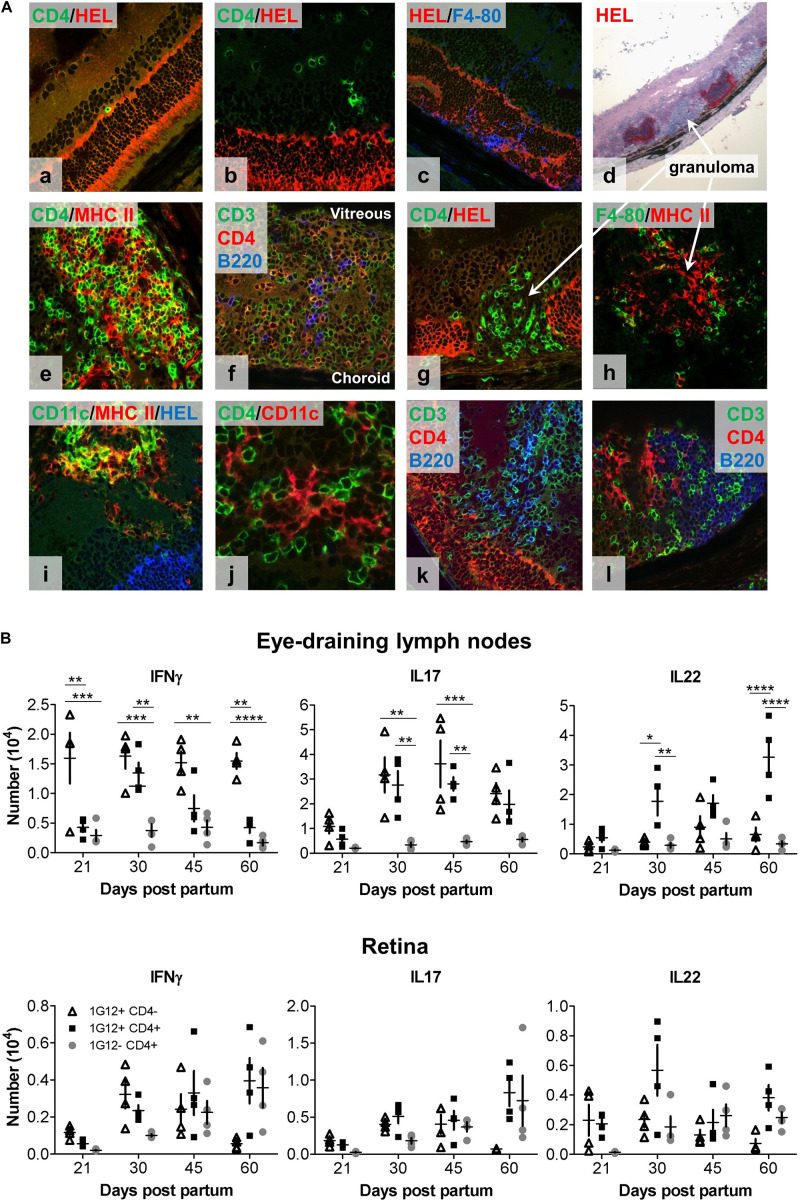
Immunohistology and intracellular cytokine analysis (IFNγ, IL17, and IL22) of eye-draining lymph node- (DLN) and retina-infiltrating cells over the course of spontaneous EAU in dTg HEL/TCR mice. **(A)** Representative confocal fluorescent **(a–c,e–l)** and light microscopic **(d)** immunohistochemistry of frozen retinal sections from (P24) dTg HEL/TCR mice are shown. Sections are representative of initial retinal inflammatory changes **(a–c)** and advanced retinal inflammatory damage **(d–l)**. Infiltrating cells identified include: **(a,b,f,k,l)**, CD3+CD4+ double-stained T cells, CD3+CD4- single-stained, presumed double-negative (DN) T cells; **(c,h)**, macrophages (F4/80+); **(e,i)**, MHC class II+ and CD11c+ (presumed DC); and **(f,k,l)**, B cells. In panels **(d,g,h)** granulomas are shown. In panels **(f,k,l)**, granulomas have features of tertiary lymphoid organs (TLO). Areas of retinal HEL protein expression were identified using a polyclonal HEL-specific antibody. The slides were mounted with Hydromount^TM^ Aqueous Non-fluorescing Mounting Media (National diagnostics, Hull, United Kingdom), and were examined with a Zeiss LSM510 confocal microscope (Carl Zeiss Meditec, Göttingen, Germany). **(B)** Flow cytometric analysis of intracellular cytokine expression in lymph node cell populations at different stages of EAU development. Single cell suspensions were prepared from eye-draining lymph nodes (upper panel) and retinas (lower panel), respectively (*n* = 4/age group). Flow cytometric analysis of intracellular cytokine expression by three different T cell populations (1G12+CD4-, 1G12+CD4+, and 1G12-CD4+) at different time points during evolution of EAU in dTg mice was completed. Upper panel (dTg DLN): at disease onset (P21), only small numbers of antigen-specific CD4+ cells gave a positive signal for intracellular cytokines; a gradual up to fivefold increase was found by P30. Significantly fewer IFNγ-secreting antigen-specific CD4+ T cells (declining further toward P60) than IL17+ and IL22+ cells were found. In contrast, levels of IL17+ cells were sustained in the DLN through P60. IL22 expression was also sustained and increased in CD4+ but not DN HEL-specific T cells through P60. CD4+ non-antigen-specific T cells (1G12-) were low for all three cytokines over the course of the observation. Lower panel (dTg retina): Cytokine expression by cells mirrored those changes found in the DLN except at P45 when there were proportionately more non-antigen specific IL22+CD4+ T cells found in the retina. By P60 non-antigen-specific CD4+ T cells in the retina expressed similar levels of IL22 and IL17 as antigen-specific cells, which suggests some level of bystander activation. Cells had been stimulated with 50 ng/ml PMA and permeabilized with 1 μM ionomycin for 5 h in the presence of monensin (BD GolgiStop^TM^, BD Biosciences, Oxford, United Kingdom). The cells were surface labeled for CD4 and 1G12 (3A9 TCR) followed by intracellular cytokine labeling for IFNγ, IL17 and IL22. 1 × 10^5^ events/sample were acquired on a BD LSR II flow cytometer. Data were analyzed for each specific time point using one-way ANOVA and Tukey’s Multiple Comparison *post hoc* Test with *p < 0.05, **p < 0.01, ***p < 0.001, ****p < 0.0001 on a 95% level of confidence. Cell numbers are the average of 4 pairs of retina or DLN, i.e., cell count/pair. 1 × 10^5^ total events were recorded. Total numbers provided were extrapolated based on total cell counts, determined using a Coulter cell counter prior to sample processing.

Immuno-histological examination of the retina provided further insight into the behavior of DN vs. CD4+ T cells in the retina. As shown by fundoscopy (Figure 1A), EAU is characterized by patches of focal inflammation which on histology present as focal granulomas separated by areas of normal retina (Figure 1B). However, by immunohistochemistry, apparently normal retina is initially infiltrated by small numbers of single CD4+ T cells in the inner and outer nuclear layers in contact with HEL+ photoreceptor membrane (Figure 2Aa,b). Damage to the outer segments was associated with F4/80+ macrophage infiltration into the subretinal space (Figure 2Ac), a characteristic early sign of EAU (23). With more severe disease, large swathes of retina had entirely lost HEL protein expression and were replaced by dense granuloma formations, while the neighboring less infiltrated retina remained HEL+ (Figure 2Ad). Triple confocal staining revealed that inflammatory granulomas contained several cell types including CD3+CD4+ dual-staining T cells, CD3+CD4- single-staining T cells (presumed DN cells), F4/80+ macrophages, CD11b+ and CD11c+ myeloid cells and B220+ B cells, and had features of TLO (24) (Figure 2Ae–l). Multiple granulomas were typically found in a single section of retinal tissue, the number and extent increasing with time. Closer examination revealed the presence of MHC Class II++ CD11c++ presumed DC (25) in the interstices of the TLO-like masses, and in close contact with many CD4+ T cells (Figure 2Aj). In contrast, CD3+CD4- (DN) T cells appeared to congregate in B cell-rich areas (Figure 2Ak,l). In summary, inflamed retinas of dTg mice presented with granulomas closely resembling TLO containing an abundance of diverse pro-inflammatory cell types with strong antigen-presenting activity fostering uveitogenesis.

### IL17 Is the Predominant Cytokine in CD4+ and DN HEL-Specific T Cells During EAU, CD4+ IL22-Expressing T Cells Dominate Late Stage Disease

Both Th1 and Th17 have been implicated in the pathogenesis of EAU (26, 27). We therefore evaluated the production of these cytokines during the course of EAU in the dTg mice (gating strategy for flow cytometric analysis provided in Supplementary Figures 4a–d). Since both IL22 and IL17 are produced by lymphoid tissue-inducer cells (LTi cells) involved in the generation of TLO [reviewed in Pipi et al. (28)] we further assessed IL22 production in dTg mice. In the lymphopenic dTg eye DLN, very small numbers of antigen-specific CD4+ T cells were positive for intracellular cytokines (∼0.5 × 10^4^) at the onset of disease (Figure 2B, upper panels) but gradually increased up to fivefold to peak levels at P30. IFNγ-secreting antigen-specific CD4+ T cells were significantly fewer in number than IL17+ and IL22+ cells and gradually declined by P60. In contrast, levels of the IL17+ cells in the antigen-specific CD4+ and DN T cell populations were sustained in the DLN through P60. In contrast, IL22 expression was sustained and even increased in CD4+ but not DN HEL-specific T cells through P60. CD4+ non-antigen-specific T cells (1G12-) secreted low levels of all three cytokines for the duration of the disease. Cytokine expression by cells infiltrating the retina mirrored the changes in the DLN except at P45 when there appeared to be proportionately more non-antigen specific IL22+CD4+ T cells in the retina (Figure 2B, lower panels). In addition, by P60 non-antigen-specific CD4+T cells in the retina expressed similar levels of IL22 and IL17 as antigen-specific cells, suggesting some level of bystander activation (Figure 2B, lower panel).

### HEL-Specific CD4+ T Cells Induce EAU on Adoptive Transfer but Require Prior Activation With Cognate Antigen

**FIGURE 3:**
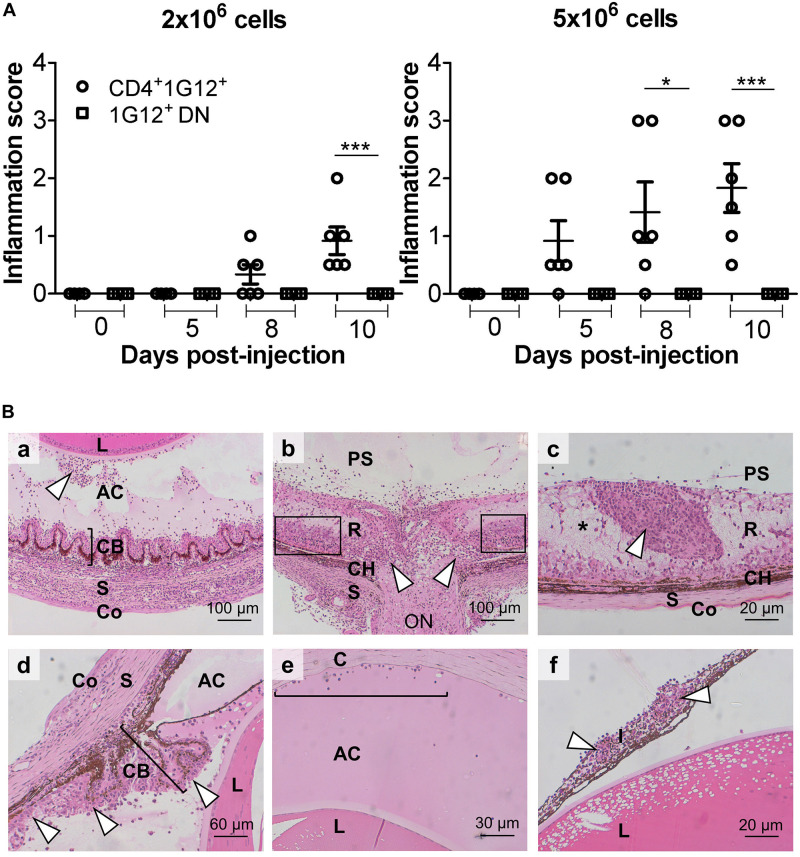
Adoptive transfer of HEL protein-activated 3A9 CD4+ T cells but not DN T cells induces severe EAU in IRBP:HEL sTg mice. **(A)** Antigen-specific CD4+ T cells and DN T cells were sorted from P50-70 3A9 TCR mice, labeled with CFSE proliferation dye and incubated with HEL protein (1 μM) for 72 h. Fundus images of sTg mice were taken, and retinal appearance clinically scored before adoptive transfer (Tx) at baseline (0; P20), and then at d5 (P25), d8 (P28) and on d10 (P30) after Tx (*n* = 6/time point). 2 × 10^6^ CD4+ cells induced EAU within 8 days while 5 × 10^6^ CD4+ cells induced severe EAU within 5 days of Tx. Adoptive transfer of DN CD3+ cells did not induce EAU despite being activated by HEL antigen even with doses of 10 × 10^6^ cells (data not shown). Data were analyzed for each time point using one-way ANOVA and Tukey’s Multiple Comparison *post hoc* Test with *p < 0.05, ***p < 0.001 on a 95% level of confidence. **(B)** Representative images of heamatoxylin and eosin (H&E) stained sections from adult (P45-P60) sTg IRBP:HEL mouse eyes, 8 days following Tx of HEL-activated CD4+ T cells from 3A9 TCR mice of the same age. Adoptive transfer induced a severe, multifocal panophthalmitis (17) **(a)** Cyclitis with “plastic” infiltrate in the vitreous and cellular precipitates on posterior lens surface (arrowhead). **(b)** Central retinal vasculitis and optic nerve destruction with granuloma plus some surrounding intact retinal tissue remaining (boxes). **(c)** Tertiary lymphoid organ (TLO; arrowhead) surrounded by edematous and necrotic retinal tissue (pale staining region; asterisk) **(d)** Inflammation of the ciliary body (bracket) (cyclitis) and posterior chamber (arrowheads) with plastic exudate in the anterior chamber. **(e)** Plastic deposit in the anterior chamber with keratic precipitates adherent to the corneal endothelium (bracket). **(f)** Massive cell infiltration in iris (iritis) with two granulomas (arrowheads). R, retina; CH, choroid; S, sclera; C, cornea; ON, optic nerve; AC, anterior chamber; L, lens; I, iris; PS, posterior segment; CB, ciliary body; Co, conjunctiva.

It was imperative to determine which T cell subset(s) induced EAU in dTg mice, and to test this antigen-specific CD4+ and DN T cells were separately isolated from P50-70 3A9 mice using magnetic bead technology (see Methods), further enriched, and adoptively transferred to P21 IRBP:HEL sTg mice. Prior analysis showed that both subsets of T cells proliferated *in vitro* in response to HEL protein, with DN cells responding less strongly (Supplementary Figure 5A). Adoptive transfer of 1–2 × 10^6^ HEL-activated CD4+ T cells was sufficient to induce EAU 8 days after transfer while 5 × 10^6^ CD4+ cells induced severe EAU 5 days after transfer both clinically (Figure 3A and Supplementary Figure 5B) and histologically (Figure 3B). Disease induction required *in vitro* activation of the T cells with HEL protein (1 μM) while non-specific activation with anti-CD3/CD28 antibody failed to induce EAU (17) (Supplementary Figure 5B). In contrast to HEL-activated CD4+ T cells, HEL-activated antigen-specific DN cells failed to induce disease at any time up to 10 days after Tx (Supplementary Figure 5B).

We next asked the central question of whether the level of retinal HEL expression determined the animals’ susceptibility to EAU (29) after Tx of activated antigen-specific CD4+ T cells. If this were the case, this finding would explain the model’s high level of antigen-specificity in EAU induction. We have shown in a companion paper series that the levels of HEL expression decline in sTg mice as retinal degeneration develops with age (12, 30). We therefore adoptively transferred 4.5 × 10^6^ CD4+ T cells to sTg mice of increasing age. Severe inflammatory disease was observed in mice of 3 weeks of age (P21) while minimal inflammation was found in mice aged 6–8 weeks old (P42-56) (Supplementary Figure 6A). No inflammation was observed in mice aged >8 months (>P240), and no disease at any age of mice was induced using CD3/CD28-activated antigen-specific T cells (Supplementary Figure 6B) (17). In conclusion, these data point toward a dose-related induction of EAU dependent on the level of HEL expression in the retina. A key observation was that the levels of retinal atrophy were similar in mice receiving HEL-activated T cells or anti-CD3/CD28 activated T cells indicating that the atrophic changes in this model are not antigen-specific (Supplementary Figures 6C,D).

### Lymphopenic dTg Mice Show Limited T Cell Anergy and Have Reduced Numbers of T_reg_

**FIGURE 4:**
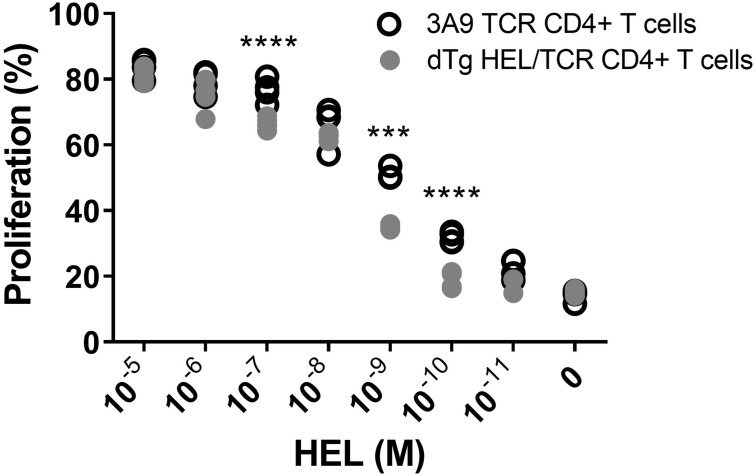
*In vitro* evidence suggests limited anergy of CD4+ cells in dTg HEL/TCR mice. CD4+ T cells isolated from 3A9 TCR or dTg HEL/TCR mice aged P50-P70 (in the presence of mitomycin C-treated antigen-presenting feeder cells from B10. Br WT mice) were incubated with decreasing concentrations of HEL protein (10^– 5^–10^– 11^ M over 72 h), and their proliferation assessed using flow cytometry to measure CFSE dilution. Limited anergy of dTg HEL/TCR CD4+ T cells (i.e., reduced specific responsiveness to HEL reflected in consistently low proliferation) was observed at and below a HEL antigen concentration of 10^– 10^ M. Significant differences were assessed using one-way ANOVA, followed by Tukey *post hoc* test with ***p < 0.001, ****p < 0.0001 on a 95% level of confidence. The experiment was independently repeated twice.

In exploring the mechanism of how T cell mediated EAU in dTg mice develops spontaneously, we had found evidence for limited T cell anergy in preceding work (11). Here, we report in detail that HEL-specific T cells in dTg mice indeed display a limited level of anergy since they proliferated *in vitro* in response to low levels of HEL, albeit less strongly than 3A9 control T cells (Figure 4). Total CD4+ cells from 3A9 and dTg mice were purified by negative selection and stimulated *in vitro* with varying amounts of HEL in the presence of mitomycin C-treated APC for three days (Figure 4). The proliferative response of dTg CD4+ T cells to high levels of HEL antigen was similar to that of 3A9 TCR controls. However, when exposed to a middle range of HEL (10^–7^ M to 10^–9^ M), the proliferative response of dTg CD4+ T cells significantly dropped compared to 3A9 CD4+ T cells (Figure 4). A significant reduction of proliferation was seen in dTg CD4+ T cells at even lower concentrations of HEL (10^–10^ and 10^–11^ M) (Figure 4). In demonstrating some level of proliferative response, dTg T cells appear different from the fully anergic T cells of similar transgenic Ins-HEL mice (21, 31) and thus have the potential to be constitutively activated *in vivo* if exposed to cognate antigen.

**FIGURE 5:**
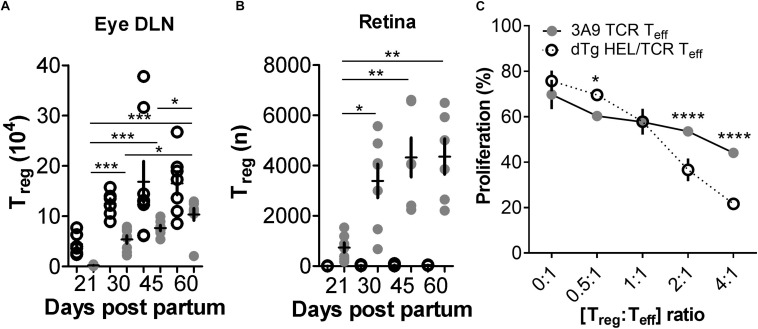
FoxP3+ T regulatory cells (T_reg_) are reduced in dTg HEL/TCR mice but accumulate in the retina in late stages of disease and have greater immunosuppressive activity than 3A9 TCR T_reg_. **(A,B)** Analysis of T regulatory cell (T_reg_) numbers in dTg HEL/TCR mice during EAU. Absolute numbers of T_reg_ in the submandibular eye DLN and retina were quantified by flow cytometry using FoxP3 and CD25 expression in CD4+ T cells. At onset of disease (P21) T_reg_ numbers were extremely low (2 × 10^3^) in the dTg DLN compared to those of 3A9 mice. No T_reg_ were detected in 3A9 retinas and effectively nil in the retinas of dTg mice at P21. As EAU developed, there was an increase in retinal T_reg_ which reached its maximum (4 × 10^3^/retina) by P45 (*n* = 6–8/age group). Data were analyzed using one-way ANOVA and Tukey’s Multiple Comparison *post hoc* Test with *p < 0.05, **p < 0.01, ***p < 0.001 on a 95% level of confidence. Cell numbers are the average of 3–4 pairs of retina or DLN, i.e., cell count/pair. 1 × 10^5^ total events were recorded. Total numbers provided were extrapolated based on total cell counts, determined using a Coulter cell counter prior to sample processing. **(C)** T_eff_ cell proliferation is significantly reduced in the presence of increasing concentrations of AgX T_reg_ compared to 3A9 T_reg_ (i.e., T_reg_ isolated from mice with EAU aged between P50 and P70; *n* = 3/genotype): dTg T_reg_ show greater suppressive ability when [T_reg_:T_eff_] ratio is [>2:1]. Data were analyzed using two-way ANOVA with *p < 0.05, ****p < 0.0001 on a 95% level of confidence.

**FIGURE 6:**
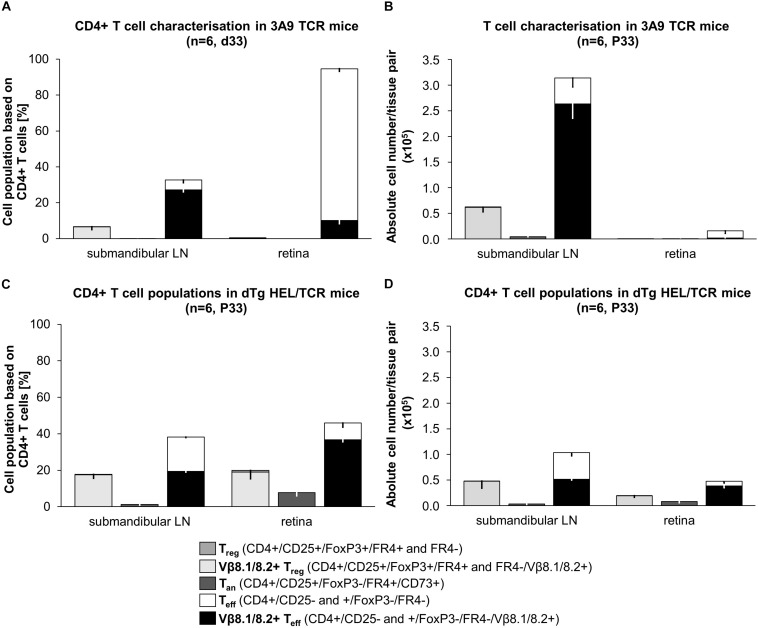
Snapshot phenotypic analysis of T cell populations in 3A9 TCR vs. dTg HEL/TCR mice at peak of EAU (P33). T cells isolated from submandibular (eye-draining) lymph nodes (LN) and retina from **(A,B)** 3A9 TCR, and **(C,D)** dTg HEL/TCR mice were evaluated by flow cytometry (BD LSR Fortessa). Anergic T cells (T_an_) are CD25+FoxP3-CD73+FR4+; regulatory T cells (T_reg_) are CD25^hi^FoxP3+FR4±; effector T cells (T_eff_) are CD4+CD25±FoxP3-. Antigen-specific cells are Vβ8.1/8.2+ (surrogate marker). Data are expressed as **(A,C)** percentages (%) relative to CD4+ T cells (dTg LN: 17.8% T_reg_, 17.5% Vβ8.1/8.2+ T_reg_, 1.2% T_an_, 38.2% T_eff_, 19.4% Vβ8.1/8.2+ T_eff_; dTg retina: 19.9% T_reg_, 19.0% Vβ8.1/8.2+ T_reg_, 7.8% Tan, 45.9% T_eff_, 36.7% Vβ8.1/8.2+ T_eff_; 3A9 TCR LN: 6.7% T_reg_, 6.6% Vβ8.1/8.2+ T_reg_, 0.5% T_an_, 32.7% T_eff_, 27.2% Vβ8.1/8.2+ T_eff_; 3A9 TCR retina: 0.5% T_reg_, 0.08% Vβ8.1/8.2+ T_reg_, 0.03% Tan, 94.6% T_eff_, 10.2% Vβ8.1/8.2+ T_eff_) and **(B,D)** as absolute numbers (*n*) of CD4+ T cell populations per sample (i.e., per pair of retina or LN). Data are presented as the average of 6 pairs or retina or 6 pairs of lymph nodes (1 pair each/mouse), extrapolated based on total cell counts, determined using a Coulter cell counter prior to sample processing. Lymphopenia, in dTg mice is reflected in the comparably low absolute CD4+ T cell numbers in lymph nodes **(D** vs. **B)**. As expected, no T_reg_ or T_an_ cells are present in retinas of 3A9 mice **(A,B)**. P33 dTg HEL/TCR mice with EAU have high numbers of Vβ8.1/8.2+ T_eff_ cells in their retinas **(C,D)**. T_reg_ and T_an_ are also present in large numbers (i.e., five- to six-fold those of 3A9 TCR mice) despite the presence of severe EAU. On a 95% level of confidence, the 2-samples Wilcoxon-Mann-Whitney rank sum test confirmed significant tissue-specific differences in both percent of CD4+ cells and absolute numbers of CD4+ cells between genotype groups (submandibular LN: T_reg_% CD4+ *p*0.004, T_an_% CD4+ *p*0.003, T_eff_% CD4+ *p*0.004, Vβ+ T_eff_% CD4+ *p*0.004; T_eff_ n CD4+ *p*0.004, Vβ+ T_eff_ n CD4+ *p*0.004; retina: *p*0.004 for each of the cell populations, expressed as both% of CD4+ and absolute numbers, *n*). For ease of reading, stacked bar charts presenting means ± SD are provided (*n* = 6/genotype).

In our experiments we also found that T_reg_ numbers in dTg lymphopenic mice in the eye DLN were very infrequent (∼2 × 10^3^), and in the retina were minimal (<1 × 10^3^) at the time of disease onset (P21) (Figures 5A,B and Supplementary Table 4). We therefore re-considered the possibility that, in addition to a relative failure of T cell anergy, deletion of T_reg_ may have contributed to EAU development allowing uncontrolled expansion of uveitogenic IL17+/IL22+ or IFNγ+ T cells. During the course of disease (up to P60; end of observation) the number of T_reg_ in the DLN increased but never reached levels equivalent to non-Tg or 3A9 mice (Figure 5A). However, they appeared to be more effective in suppressing T cell proliferation *in vitro* (Figure 5C). Notably, the number of T_reg_ in the retina of dTg mice increased significantly during this period (Figure 5B; gating strategy for detection of T_reg_ provided in Supplementary Figures 7a,b) which coincided with stabilization and resolution of inflammation in the eye (Figure 1 and Supplementary Figure 2). Similar results were obtained when comparing retinas of P33 dTg vs. 3A9 TCR mice in terms of non-pathogenic cell populations. While in the eye DLN, the distribution pattern of those cell populations of interest were highly similar between the genotypes, there was a clear increase in T_reg_ and T_an_ in retinas of dTg mice, compared to their 3A9 counterparts (Figure 6).

### Limited Anergy vs. Reduced T_reg_ in EAU Pathogenesis in dTg Mice

Recent studies have suggested that generation of T_an_ and T_reg_ may under certain conditions be a reciprocal process (1, 32). Since we have found that both limited anergy and lack of T_reg_ potentially contributed to development of EAU in dTg mice, we explored the phenotype of these T cell populations at a single time point (P33) when EAU severity is approaching peak levels (Figure 1). Anergic T cells were identified as CD25+FoxP3-CD73+FR4+ cells, while T_reg_ were identified as CD25^hi^FoxP3+FR4± (33, 34) and their relative proportions within the AgX population of P33 mice with EAU and in 3A9 mice were compared (Figure 6).

Unfractionated DLN CD4+ T cells in P33 3A9 mice contain ∼33% CD25+FoxP3- effector cells, of which 83% are Vβ8.1/8.2+ [antigen-experienced (35)] (Figure 6A). Approximately 7% T_reg_ cells are also present in the DLN CD4+ T cell population, all of which were HEL-specific. In addition, there were ∼0.5% T_an_ cells indicating that there is some degree of overlap between these two populations of T cells (Figure 6A). In the P33 3A9 retina, there were few T cells (Figure 6B) probably representing contaminating intravascular cells.

Unfractionated cells in the DLN of the lymphopenic dTg mice at P33 contained a significant proportional increase in T_reg_ (up to ∼18%) as well as T_an_ cells (up to ∼2%) although the absolute number of T_reg_ and T_an_ were the same as in 3A9 mice. Proportionally there were nearly twice as many T_reg_ and T_an_ in the DLN of dTg mice compared to 3A9 mice. In the retina of P33 dTg mice, there were up to 20% T_reg_ while T_an_ accounted for 8% of the CD4+ T cell population indicating similar [T_reg_:T_eff_] and [T_reg_:T_an_] ratios as in the dTg DLN (Figures 6C,D).

### Antigen-Experienced CD4+FoxP3+ T_reg_ Arrest EAU Development in dTg HEL/TCR Mice

**FIGURE 7:**
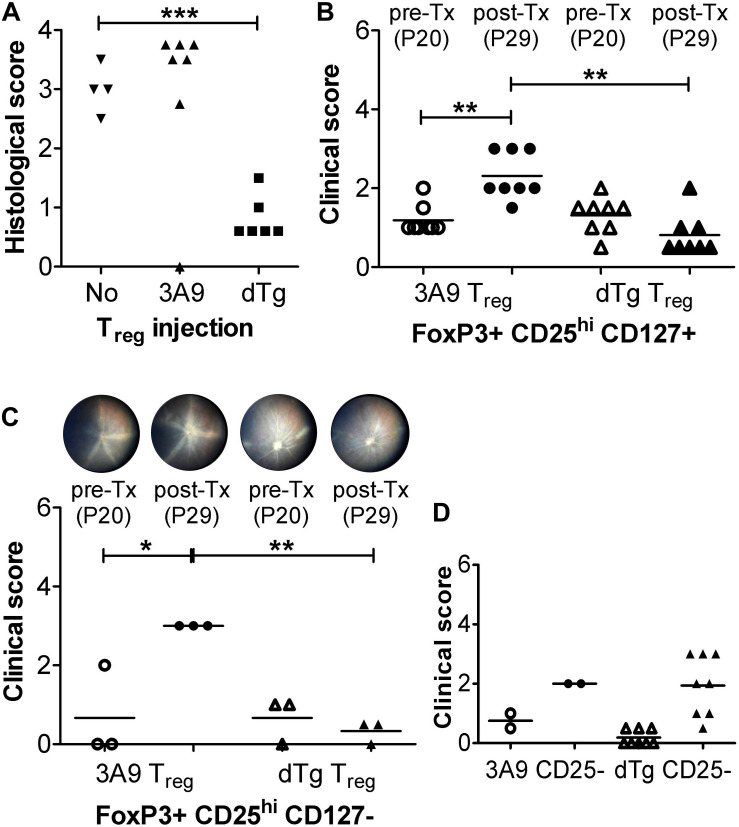
Adoptive transfer (Tx) of FoxP3+ T regulatory cells (T_reg_) arrests EAU progression. **(A)** Adoptive transfer (Tx) of unfractionated T cells (1 × 10^6^/mouse). Lymph node cells (submandibular and superficial cervical LN) were collected from adult P28-P42 3A9 TCR or dTg HEL/TCR mice and adoptively transferred (i.v.) to P21 mice (*n* = 6–7/treatment group). Control mice received no cells (“No”; *n* = 4). Eyes were taken for histology at P30. ^∗∗∗^*p* < 0.001 on a 95% level of confidence. **(B,C)** Tx (i.v.) of enriched FoxP3+CD25+ T_reg_ cells from spleen and submandibular, superficial cervical, axillary, and inguinal LN, passed through a CD4+CD25+ regulatory T cell isolation kit (see section “T_reg_ Cell Isolation”) [**B:** CD127+; **C:** CD127-; 1 × 10^6^/mouse; bead-based CD127 negative selection (18)] arrests EAU progression. T_reg_ were isolated from 3A9 TCR mice aged P50-70 (naïve T_reg_), and dTg mice of the same age (antigen-experienced T_reg_), and adoptively transferred (i.v.) to P21 dTg mice. **p < 0.01, *p < 0.05 on a 95% level of confidence. In control experiments **(D)** FoxP3-CD25-CD4+ effector cells from P50-70 3A9 controls (*n* = 2) and dTg mice of the same age (*n* = 8) were administered to P21 mice (3 × 10^6^/mouse), and their effect on EAU progression evaluated. Fundus images of P20 mice were taken on the day before Tx (pre-Tx) and repeated 9 days later (post-Tx), on P30. Eyes were assessed for inflammation using fundoscopy **(B–D)**, and histology **(A)** (see “Materials and Methods”) ^∗∗^*p* < 0.01 on a 95% level of confidence.

Our data propose that the proportional increase in T_reg_ in the retina (Figure 6B vs. **D**) might promote resolution of EAU which was found to stabilize at ∼P44-59 and gradually burn out (Figure 1 and Supplementary Figure 2). We therefore asked whether Tx of AgX T cells, i.e., cells from mice at peak to late stages of disease/disease resolution, which would contain increased numbers of T_reg_ (∼18–20%) (Figure 6), might prevent development of disease if they were administered at time of disease onset (P20). To this end, initial pilot experiments were performed with unfractionated T cells and histological assessment only (Figure 7A). While Tx of naïve (3A9) T cells had no effect on disease progression, cells from dTg mice (AgX cells) completely prevented development of EAU (Figure 7A). Importantly, the transferred cells had not been activated by HEL antigen *in vitro* prior to Tx. Next, Tx experiments were performed using purified cell populations and clinical grading of EAU. Adoptive transfer of both FoxP3+CD25^hi^CD127+ and FoxP3+CD25^hi^CD127- T_reg_ from dTg mice but not from 3A9 mice prevented development of EAU and in some cases reversed disease (Figures 7B,C and Supplementary Figure 8). In stark contrast, Tx of FoxP3-CD4+CD25-CD127+ T cells did not significantly alter progression of disease (Figure 7D). Phenotypic analysis of the T_reg_ population within the immunosuppressive antigen-experienced T cells is summarized in Table 1. The representative data shown attest to a FoxP3+, non-Tr1 T_reg_ type.

**TABLE 1:** Phenotype of immunosuppressive, antigen-experienced T_reg_.

Pre-CD4+ enrichment	

Marker	Percent within T_reg_ cells (CD4+CD25+FoxP3+)
Eomes+	0.5
Helios+	70.0
CD127+	15.0
LAG3+	1.0

**Post-CD4+ enrichment**	

Eomes+	0.0
Helios+	97.0
CD127+	0.8
LAG3+	0.3

*Flow cytometric characterization of CD4+CD25+FoxP3+ T_reg_ cells obtained from dTg HEL/TCR mice, pre- and post-magnetic bead CD4+ enrichment. Representative data are provided.*

## Discussion

We report here new data in a previously described model of spontaneous experimental autoimmune uveoretinitis (EAU) generated by cross-breeding IRBP:HEL sTg mice (sTg) with 3A9 TCR mice to produce dTg HEL/TCR (dTg) mice (11). In a companion paper series (12, 30), we showed that insertion of the HEL gene in B10. BR wild type mice to create the sTg mice, induces a non-inflammatory, mild to moderate retinal degeneration with photoreceptor shortening. This is associated with a relative reduction in the amount of IRBP in the retina, an essential retina-specific protein for normal photoreceptor health (36). The retina is otherwise normal particularly in the early post-natal period and sTg mice do not develop uveitis.

In the present study, dTg mice develop signs of retinal inflammation (vasculitis and granulomatous disease) in a patchy focal distribution, at several sites in the fundus, as early as P20 which progresses rapidly to involve the entire retina. There is slight variability with regard to the time of onset of the disease and ultimate severity, but by P44 100% of mice have severe (grade 3–4) signs of EAU. In addition, there is associated retinal atrophy which culminates in extensive loss of retinal tissue and in some mice (30% of mice by P90) progresses to involve the entire globe (phthisis). The disease thus resembles severe progressive chronic ocular inflammatory disease in humans (autoimmune uveitis) in several aspects including long-term globe shrinkage (37).

3A9 TCR mice contain around 70% HEL-specific CD4+ T cells in the peripheral lymphoid tissue, >90% of which express a Vβ8.2 TCR and can be directly identified using a specific antibody against Vβ8.1/8.2 (11, 38). Flow cytometric analysis of eye DLN cells in dTg mice indicated there was extensive thymic deletion of T cells in the early stages of disease (P20), which affected HEL-specific CD4+ T cells particularly. Peripheral lymphoid tissues were populated predominantly with a second HEL-specific population of DN T cells (CD3+CD4-CD8-) and remained so for at least 60 days when this part of the study was concluded. In the eye DLN, CD4+ HEL-specific T cells increased in frequency as the disease progressed but less so in non-eye DLN (data not shown). Since HEL antigen is expressed in the thymus (11) and in the peripheral lymphoid tissues (29) in sTg mice, HEL-specific CD4+ T cell deletion in dTg mice is predictable. In similar models, skewing of the peripheral T cell population toward DN T cells is well recognized and considered to be characteristic of some dTg TCR models (39).

DN T cells were also the most frequent T cell in the retinal cell infiltrate at the onset of inflammation. However, during progression of the disease they became less frequent while the numbers of CD4+ HEL-specific T cells remained constant. In addition, IL17 and IL22-expressing CD4+ HEL-specific T cells were more frequent and consistent than DN and non-HEL-specific endogenous T cells. Both CD4+IFNγ+ and IL17+ T cells are recognized as pathogenic in conventional models of EAU [reviewed in Lee et al. (40)] and if pathogenicity can be attributed to specific cytokines, IFNγ+ and IL17+ DN cells in this model would certainly be considered pathogenic. However, in the dTg HEL/TCR mouse reported here, the predominant CD4+ T cells which are pathogenic were IL17 producers, as revealed by Tx experiments to sTg mice. Despite the fact that both the CD4+ T cells and DN T cells respond to HEL protein *in vitro* this is particularly remarkable. In fact, endogenous non-HEL-specific T cells may play a more significant role than DN T cells in the dTg model, since they become the dominant cell in the late stages of disease, and Rag-/- dTg mice do not develop EAU (11). This also raised the central question of whether the amount of HEL antigen expressed in the retina influences the degree of the inflammatory response it triggers. We have shown that sTg mice, which develop an age-related photoreceptor degeneration with reduced levels of HEL expression (12), become less susceptible to HEL-activated 3A9 CD4+ T cell-induced EAU. The precise role the DN T cells play in this model is not clear. The CD4 molecule is known to significantly amplify T cell responses [reviewed in Huppa et al. (41)] which may partly explain the lack of DN T cell pathogenicity *in vivo*, although DN cells respond well to HEL *in vitro*. In addition, the process of generation of transgenic αβTCR appears to disrupt later expression of γδTCR as well as the CD8 molecule, and it can be surmised that it also affects proper expression of the CD4 molecule (42, 43). Other possibilities can be envisaged. Previous studies have suggested that a subset of CD3+CD4- T cells may actually have an immunoregulatory role (44) but it may also be that, in the current model, the frequency of DN T cells is simply a response to the vacated lymphopenic space (45) generated by the extensive thymic deletion.

The data thus confirm that the pathogenic T cell in this model is the CD4+ T cell. In addition to IFNγ and IL17, CD4 T cells, but not DN T cells, expressed increasing levels of IL22 into the late stages of the disease in the DLN. In the retina, IL22 was expressed by both CD4+ HEL-specific T cells and endogenous CD4 T cells, but not DN cells. This correlated with the immunohistochemical change from a pauci-cellular infiltration of CD4+ T cells and myeloid cells in the photoreceptor layer to the development of large full thickness granulomas containing many cell types, including B cells, with the characteristics of tertiary lymphoid organs (TLO). These are considered to be sites where active local antigen presentation by DC occurs in the inflamed tissue and have been previously observed in a Tg IRBP-TCR model of EAU (46). IL22 is known to be involved in lymphoid tissues inducer type cells (47) *in situ* and both IL22 and IL17 are implicated in the formation of TLO (15, 48). These data fit with IL22+IL17+CD4+ T cells being primarily pathogenic in this model with a secondary contribution from the endogenous T cell populations. Most recently, however, IL22 has been shown to have a regulatory effect on retinal inflammation when administered locally, indicating that its precise role in this model is not yet defined (49).

The mechanism whereby tolerance is broken, and spontaneous autoimmunity develops in dTg mice was attributed, in a related preceding study, to reduced T cell anergy (11). The suggestion that lymphopenia might drive the susceptibility to antigen-specific EAU is supported by previous work from Gregerson’s lab who showed that adoptive transfer of beta-galatosidase TCR specific (bgalTCR) T cells, even when activated *in vitro* prior to transfer, failed to induce EAU in mice expressing bgal under control of the promoter for retinal arrestin (arrbgal mice). In addition, bone marrow chimeric adult arrbgal mice failed to develop EAU. However, mice rendered lymphopenic developed EAU when they were adoptively transferred with bgal+ TCR T cells depleted of T_reg_ (CD25+ T cells). Thus, the combination of lymphopenia-induced reduction in the numbers of endogenous T_reg_ plus depletion of exogenous T_reg_ from the adoptive T_eff_ cell population, allowed the development of severe EAU (50). The data in the current paper support this view since spontaneous disease develops when the mice are profoundly lymphopenic, and particularly in the T_reg_ compartment (Figure 5). The current work also takes it to the next level by showing that adoptive transfer of a population of antigen-experienced, T_reg_-containing CD4+ T cells, can prevent lymphopenia-associated spontaneous EAU in dTg HEL/TCR mice.

We also explored T_reg_ function *in vitro* and while there was some evidence for greater proliferation of CD4+CD25+ T_reg_ from dTg mice, T_reg_ measured in the thymus and spleen were not significantly different nor could we generate any convincing *in vivo* evidence for a T_reg_ effect using ML5 recipients (11). Hence, the question of an additional defect in T_reg_ function in this model was left open. In the present study, we revisited this question. We first confirmed that CD4+ T cells from dTg mice with EAU were not fully anergic as has been reported, for instance, for Ins-HEL CD4+ T cells (21) although they responded less well to low doses of HEL *in vitro* than 3A9 CD4+ T cells. We next re-explored the role of T_reg_. As for the overall T cell population, CD4+CD25+FoxP3+ T_reg_ underwent extensive thymic deletion in the early postnatal period and although they gradually increased in the eye DLN to P60, they were consistently reduced in number compared to non-Tg WT mice (data not shown) and to 3A9 control mice (Figure 5A). It thus appeared that both limited anergy and reduced T_reg_ numbers contributed to development of autoimmunity in this lymphopenic model of EAU. Recently it has been shown that there is a reciprocal relationship between T_reg_ (CD25^hi^FoxP3+FR4±) and T_an_ (CD25+FoxP3-CD73+FR4+) cells (32, 33). We therefore explored this relationship in the dTg model at a single time point of peak disease and found that while the proportions of T_reg_ and, less so, of T_an_ in the DLN of dTg mice were greater than in the DLN of 3A9 mice, the absolute numbers were similar in both DLN. Unlike T effector cells (CD25-/+) they were 100% HEL-specific. In contrast, in the inflamed retina of dTg mice, the relative and absolute proportions of T_an_ were greater than in the DLN, suggesting that there was a higher trend toward T_an_ than T_reg_ generation at the site of tissue damage. Which of these two populations is the more effective in terms of suppressing disease is unclear, but our *in vitro* studies indicated that, in the context of T_reg_, antigen-experienced dTg T_reg_ are more effective than naïve 3A9 T_reg_.

Gregerson’s group have made a strong case for T_reg_ generation locally, based in part on the lack of bgal expression in the thymus as well as local depletion studies using DTR/GFP mice crossed to the arrbgal mice (51). Our data support this concept to some extent, as we suggest that T_eff_ may become T_an_ and convert to T_reg_ *in situ* in the retina during EAU progression (Figure 6). However, the development of spontaneous EAU in the current model is likely to be mostly due to a failure of thymic T_reg_, not only because HEL is expressed in the thymus (11) but because of the profound lymphopenia and virtual absence of T_reg_ from the onset of disease at P21 (Figure 5).

We therefore decided to directly compare dTg antigen-experienced T_reg_ with 3A9 T_reg_ *in vivo*. We hypothesized that the small numbers (∼0.7 × 10^3^) which were detected in the retina at P21 and gradually increased to ∼4 × 10^3^ at P60 were insufficient to prevent disease. Accordingly, we adoptively transferred AgX unfractionated T cells to P21 mice before the onset of disease and showed by histology at P30 that spontaneous development of EAU was arrested. Further experiments using FoxP3+CD25^hi^CD4+ T_reg_ isolated from > P50 3A9 mice (naïve) or from > P50 dTg (AgX), showed that 3A9 T_reg_ were ineffective while AgX T_reg_, not only halted disease progression but reversed the pathological changes (retinal vasculitis) in some cases. Further purified T_reg_ to deplete CD127+ cells were equally effective in preventing EAU development while Tx of CD25- T cells was ineffective. The T_reg_ enriched populations were negative for Eomes and were 97% Helios positive indicating that no Tr1 cells were included in the T_reg_ sample and that a significant proportion were (natural) nT_reg_ (52).

These data strongly suggest a dual mechanism of immunological tolerance in the retina, including regulation of autoreactive T cells both by T_an_ as well as by T_reg_. They also point toward a therapeutic role for T_reg_ in immune-mediated diseases. There is considerable evidence for T_reg_ control of autoimmunity in many systems and their potential use in clinical autoimmune disease is under intensive investigation [reviewed in Esensten et al. (53)]. To generate sufficient numbers, T_reg_ may be expanded simply by administering IL2, an essential cytokine for T_reg_ growth (54), by accessory cell-based therapeutic regimes including tolerogenic DC [reviewed in Takenaka and Quintana (55)], or potentially by folate treatment (34). T_reg_-specific upregulation of the folate surface receptor FR4 is commonly accepted, and likely expressed under the control of the FoxP3 transcription factor (34). It has been shown that T_reg_ require comparably large amounts of folate to stabilize their suppressive phenotype and retain high proliferative capacity *in vivo* (56). Conversely, CD4+ T cell populations, when depleted of nT_reg_ by functional blockade of the FR4 receptor, induce autoimmune disease after Tx to nude mice (34). Thus, signaling *via* the folate receptor appears to be critical for immune regulation and its role in reciprocal conversion of T_reg_ to T_an_ may be of considerable relevance to the application of T_reg_ as a cell therapy for autoimmune disease. In the context of uveitis, T_reg_ are known to be involved in the control of experimental models (57) and appear to underpin the mechanisms of action of a number of standard clinical regimes (40). Control of uveitis by direct administration of T_reg_ has been shown for both systemic (16) and local application (58, 59).

Despite these promising reports, several questions require to be answered before T_reg_ cell therapy can become part of the therapeutic armamentarium. These include the effectiveness of antigen-specific vs. polyclonal T_reg_ (58), the “plasticity of T_reg_” (60), the combined contribution of T_an_ and T_reg_ (this paper) and their site of action. In this study, the data support a local effect of T_reg_ as uveitis-suppressing T_reg_ appear to accumulate preferentially in the inflamed retina possibly *via* T_an_ (or *vice versa*), and previous work has shown the effectiveness of direct intravitreal inoculation of T_reg_ (59). On the other hand, the data in this study also suggest that AgX T cells are more effective than naïve T cells, but it is possible that non-specifically activated T_reg_ may be equally effective. Hence, it remains to be clarified whether “spontaneous” uveitis as it occurs in this model of EAU is triggered by a quantitative imbalance in cell populations (e.g., [T_reg_:T_eff_]; [T_reg_:T_an_]) or if, qualitatively, “antigen-experienced” T_reg_ are essential and if so, whether tissue-specific antigen is necessary to control organ-specific disease. These experiments are in progress.

In conclusion, the model described in this report is reproducible, reliable and valuable for pre-clinical studies of therapies for uveitis and it opens up new avenues for research into mechanisms of tolerance in autoimmunity.

## Data Availability Statement

The datasets generated for this study are available on request to the corresponding author.

## Ethics Statement

The animal study was reviewed and approved by University of Aberdeen, Ethical Review Committee, University of Aberdeen, King’s College, Aberdeen AB24 3FX, United Kingdom.

## Author Contributions

HW, LK, RC, and JF: study design. Y-HL, CM, KM, KK, CC, IK, DR, LK, and RC: *in vivo* and *in vitro* work/generation of data and materials. Y-HL, CM, KM, and KK: statistical analyses. Y-HL, CM, and JF: manuscript conceptualization and writing and formatting. All authors have critically revised the manuscript and agreed to its submission in the presented form.

## Conflict of Interest

The authors declare that the research was conducted in the absence of any commercial or financial relationships that could be construed as a potential conflict of interest.
