# Supplementary material for: Treatment With FoxP3+ Antigen-Experienced T Regulatory Cells Arrests Progressive Retinal Damage in a Spontaneous Model of Uveitis
Source: Front Immunol. 2020 Sep 4;11:2071. doi: 10.3389/fimmu.2020.02071 (PMC7498671; doi:10.3389/fimmu.2020.02071)
Supplement: Supplementary file 1 [file Presentation_1.pdf]

# Treatment with FoxP3+ Antigen-Experienced T Regulatory Cells Arrests Progressive Retinal Damage in a Spontaneous Model of Uveitis

Yi-Hsia Liu[a, b, †], Christine Mölzer[a, †], Kimmo Makinen[a, c], Koju Kamoi[a, d], Clare L. C. Corbett[a, e], Izabela P. Klaska[a, f], Delyth M. Reid[a], Heather M. Wilson[a], Lucia Kuffová[a], Richard J. Cornall[g], John V. Forrester[a, *]

[a]*University of Aberdeen, Institute of Medical Sciences, Foresterhill, Aberdeen, AB25 2ZD, UK.*

[b]*Present address: Wolfson Wohl Cancer Research Centre, Institute of Cancer Sciences, University of Glasgow, Bearsden, G61 1BD, UK.*

[c]*Present address: Human Health, Novozymes A/S, Krogshøjvej 36, 2880 Bagsvaerd, DK.*

[d]*Present address: Department of Ophthalmology and Visual Science, Graduate School of Medical and Dental Science, Tokyo Medical and Dental University, Tokyo, JP.*

[e]*Present address: School of Life Sciences, University of Nottingham, East Drive, Nottingham NG7 2TQ, UK.*

[f]*Present address: Institute of Ophthalmology, University College London, 11-43 Bath Street, London, EC1V 9EL, UK.*

[g]*MRC Human Immunology Unit – MRC Weatherall Institute of Molecular Medicine, University of Oxford, Oxford, OX3 9DS, UK.*

[†]*These authors (in alphabetical order) have contributed equally to this work and share first authorship.*

[*]**Correspondence:**

Prof. John V. Forrester
University of Aberdeen, Institute of Medical Sciences, Foresterhill, Aberdeen, AB25 2ZD, UK.
E-mail: j.forrester@abdn.ac.uk
Phone: +44 1224 437507
Fax: +44 1224 437506

## Supplementary Data

| Feature | | | Score | | | | | |
|---|---|---|---|---|---|---|---|---|
| | | | 0 | 1 | 2 | 3 | 4 | 5 |
| **A** | **Infiltrate** | Number | < 5 | > 5 | > 5 | > 1 | | |
| | | Size | Small | Small | Medium | Large | | |
| **B** | **Vasculitis** | | - | Mild (less than 50 % of vessels affected) | Mild (more than 50 % of vessels affected) | Moderate | Severe | |
| **C** | **Haemorrhage** | | - | - | - | - | Present | |
| **D** | **Vitreous haze** | | - | Minimal | Minimal | Mild | Moderate | Severe |
| **E** | **Retinal detachment** | | - | - | - | - | - | Present |

**Supplementary Table 1.** *Scoring system used for clinical assessment of the severity of retinal inflammation using fundoscopy.*

| Score | Feature |
|---|---|
| **0** | Normal fundus appearance |
| **0.5** | Occasional “drusen-like” white lesions |
| **0.75** | Multiple drusenoid deposits plus < 3 small/medium sized atrophic patches |
| **1** | < 5 medium/large atrophic patches |
| **2** | < 10 medium/large atrophic patches |
| **3** | Medium/large atrophic patches involving ~ 50 % of fundus |
| **4** | Extensive chorioretinal contiguous patches of atrophy involving > 75 % of retina |

**Supplementary Table 2.** *Scoring system used for clinical assessment of the severity of retinal atrophy using fundoscopy.*

# Supplementary Figure 1 a

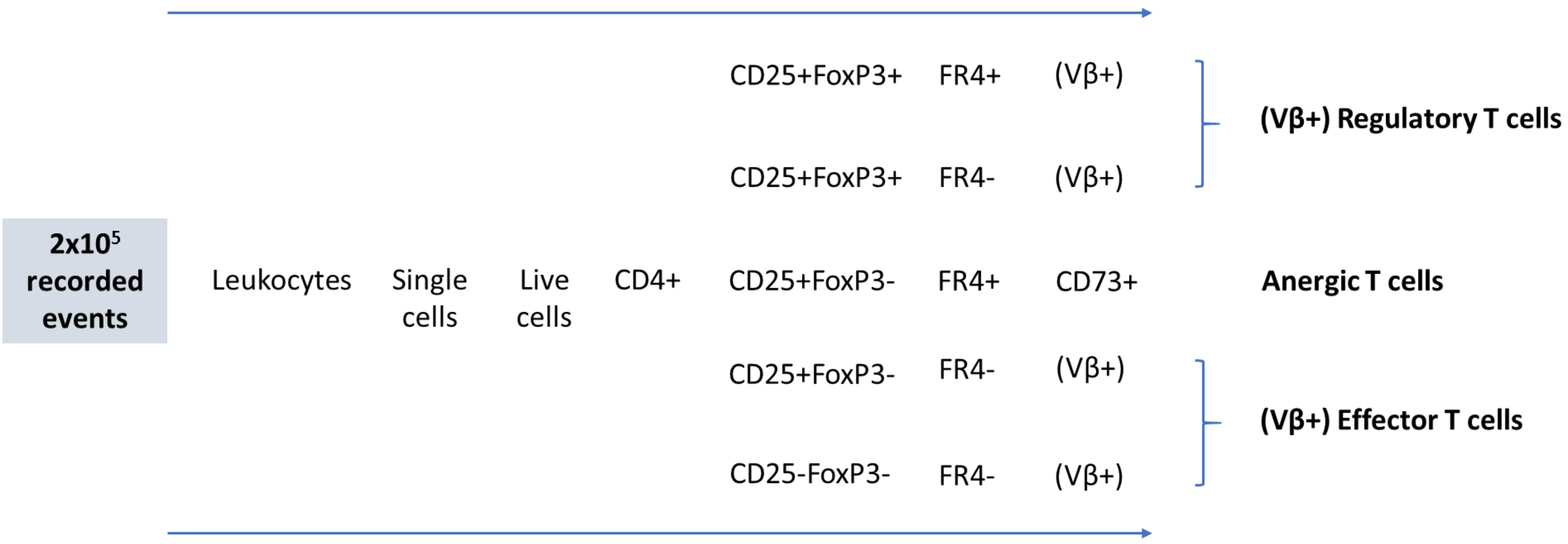


**Supplementary Figure 1 a.** *Basic flow cytometry gating strategy for phenotypic characterisation of cell populations in retinas and submandibular (eye-draining lymph nodes) of dTg HEL/TCR and sTg 3A9 TCR mice.* The gating strategy (used in Figure 6) for T regulatory ($T_{reg}$), Vβ8.1/8.2+ $T_{reg}$, anergic T cells ($T_{an}$), T effector ($T_{eff}$) cells, and Vβ8.1/8.2+ $T_{eff}$ cells is presented.

# Supplementary Figure 1 b

## Retina

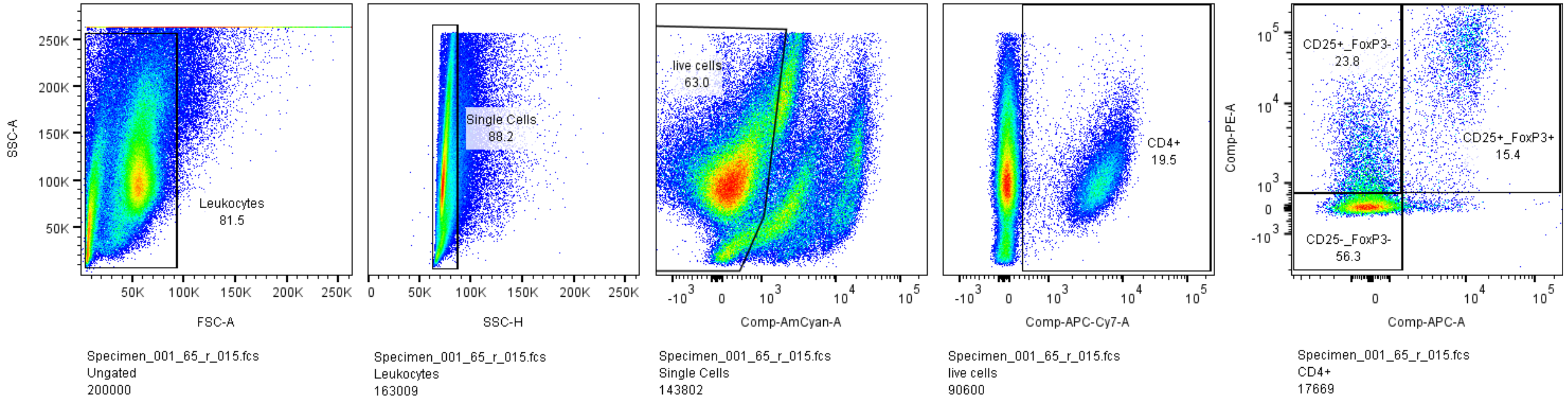


## Eye-draining lymph nodes

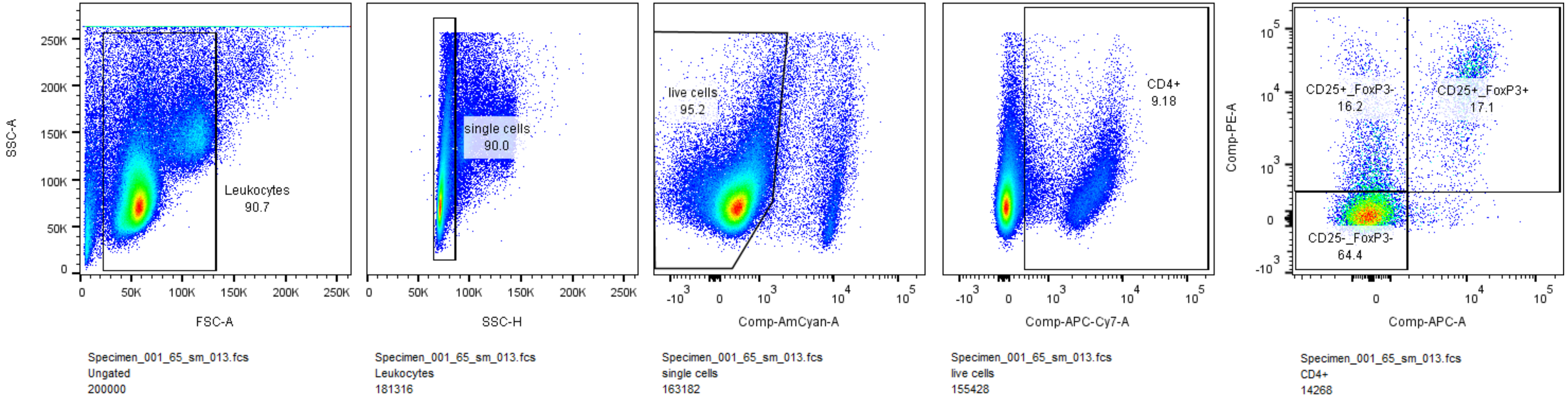


**Supplementary Figure 1 b.** *Basic population-based flow cytometry gating strategy for phenotypic characterisation of cell populations in retinas and submandibular (eye-draining lymph nodes) of dTg HEL/TCR and sTg 3A9 TCR mice.* This strategy applies to all populations of interest, as outlined in Supplementary Figure 1 c - f. Gating was based on respective controls (unstained for live/dead; individual FMO for each fluorochrome < 1 % of parent). Gate percentages refer to respective parent gates, as indicated in each plot legend (absolute cells in parent gate given). The panel shows data from a dTg HEL/TCR at P33). Upper panel shows cells in retina, lower panel in eye-draining lymph nodes. This gating strategy was applied on the data presented in Figure 6.

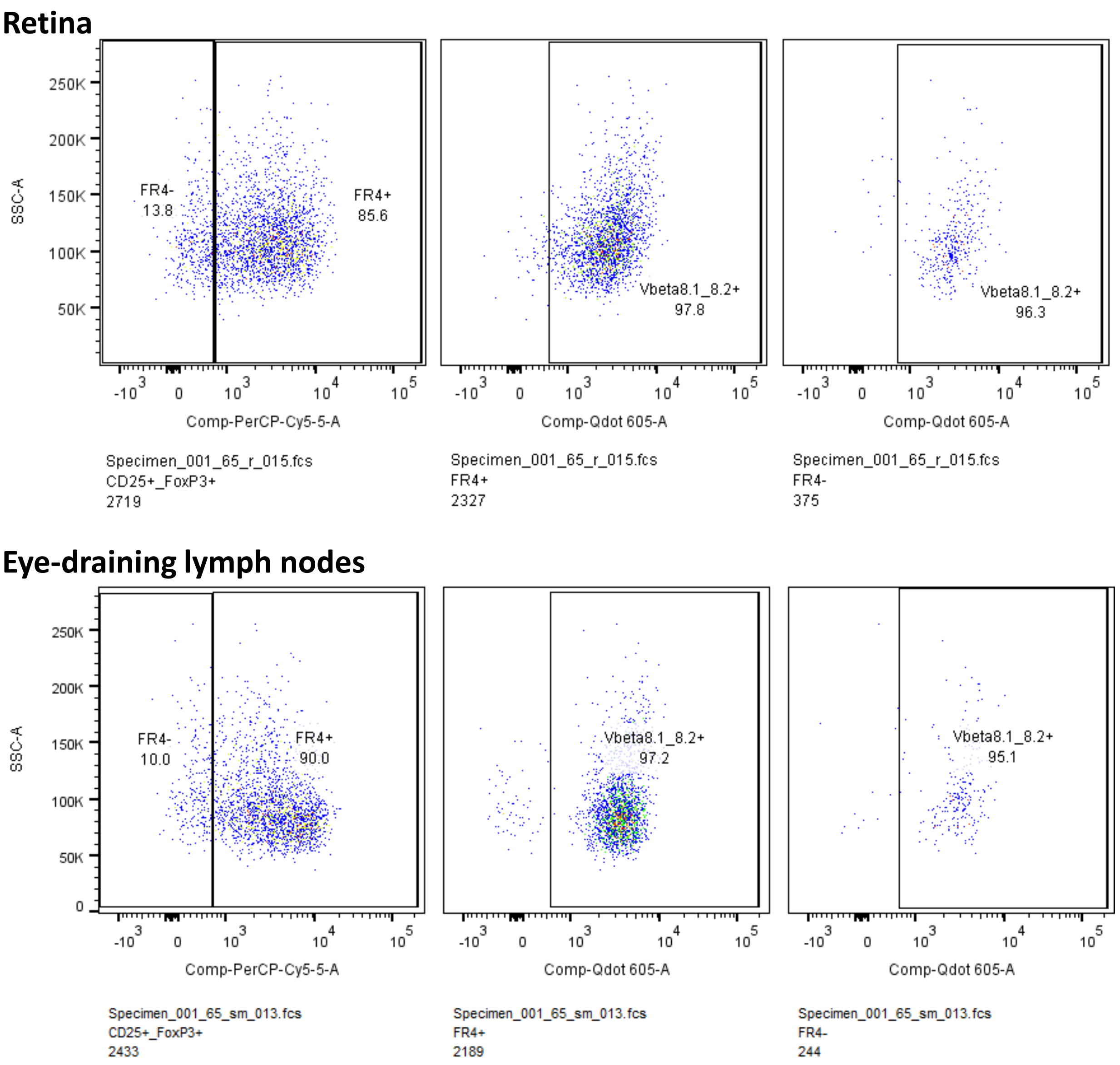


**Supplementary Figure 1 c.** *Population-based flow cytometry gating strategy for phenotypic characterisation of T regulatory cells ($T_{reg}$) and Vβ8.1/8.1+ $T_{reg}$ in P33 dTg HEL/TCR retina (upper panel) and eye-draining lymph nodes (lower panel).* $T_{reg}$ cells were defined as CD4+ CD25+ FoxP3+ FR4-/+. Vβ8.1/8.2+ $T_{reg}$ cell gating is also shown. Gate percentages refer to the cell number contained in the respective parent gate, as provided in each plot legend. This gating strategy was applied on the data presented in Figure 6.

# Supplementary Figure 1 d

## Retina

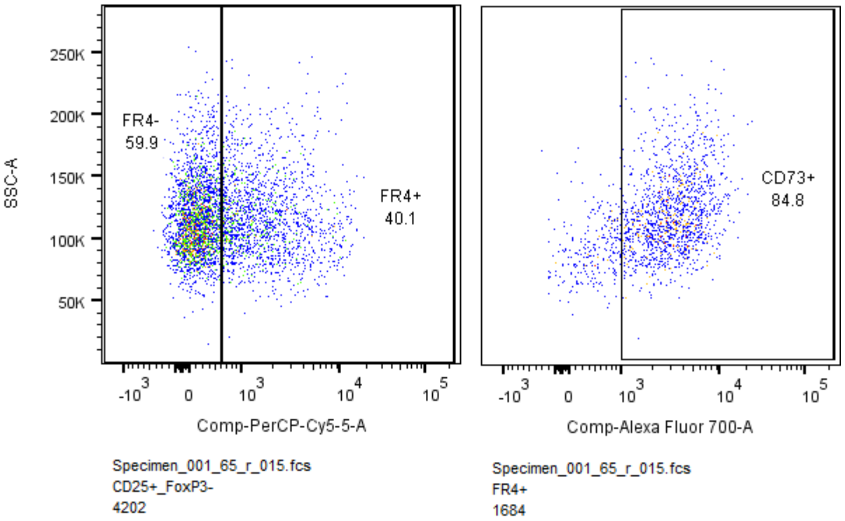


## Eye-draining lymph nodes

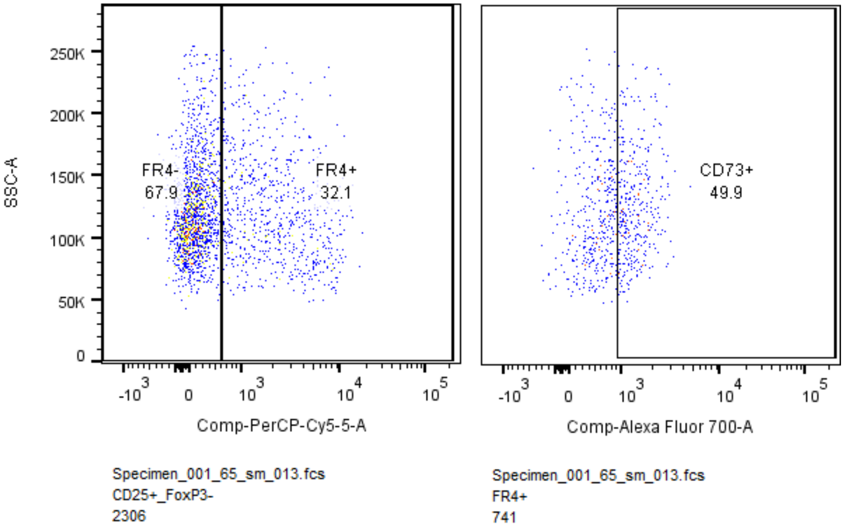


**Supplementary Figure 1 d.** *Population-based flow cytometry gating strategy for phenotypic characterisation of anergic T cells ($T_{an}$) in P33 dTg HEL/TCR retina (upper panel) and eye-draining lymph nodes (lower panel).* $T_{an}$ cells were defined as CD4+ CD25+ FoxP3- FR4+ CD73+. Gate percentages refer to the cell number contained in the respective parent gate, as provided in each plot legend. This gating strategy was applied on the data presented in Figure 6.

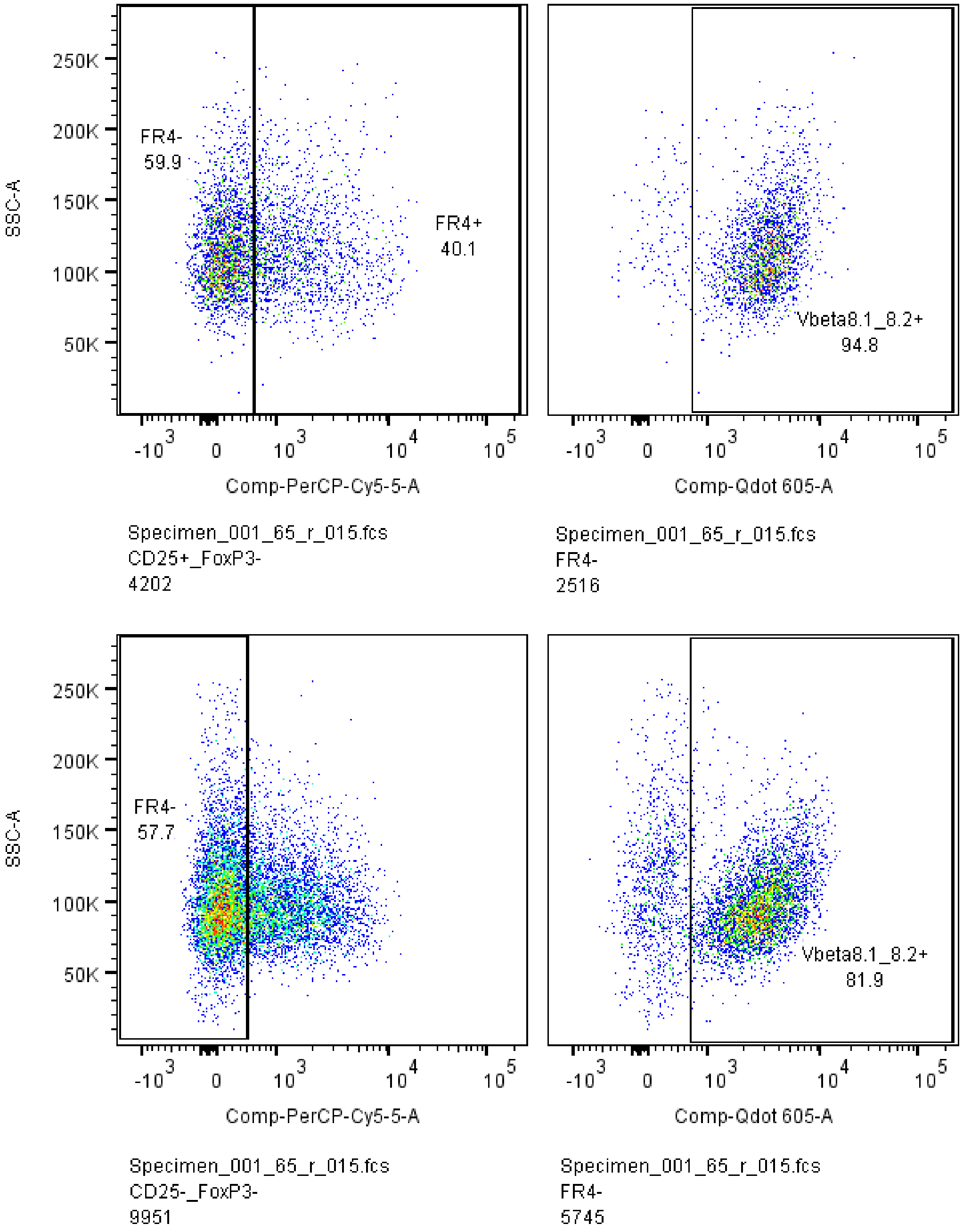


# Retina

**Supplementary Figure 1 e.** *Population-based flow cytometry gating strategy for phenotypic characterisation of T effector cells ($T_{eff}$) in P33 inflamed dTg HEL/TCR retina.* $T_{eff}$ cells were defined as CD4+ CD25-/+ FoxP3- FR4-. Gating for Vβ8.1/8.2+ $T_{eff}$ cells is also provided. Gate percentages refer to the cell number contained in the respective parent gate, as provided in each plot legend. This gating strategy was applied on the data presented in Figure 6.

# Supplementary Figure 1 f

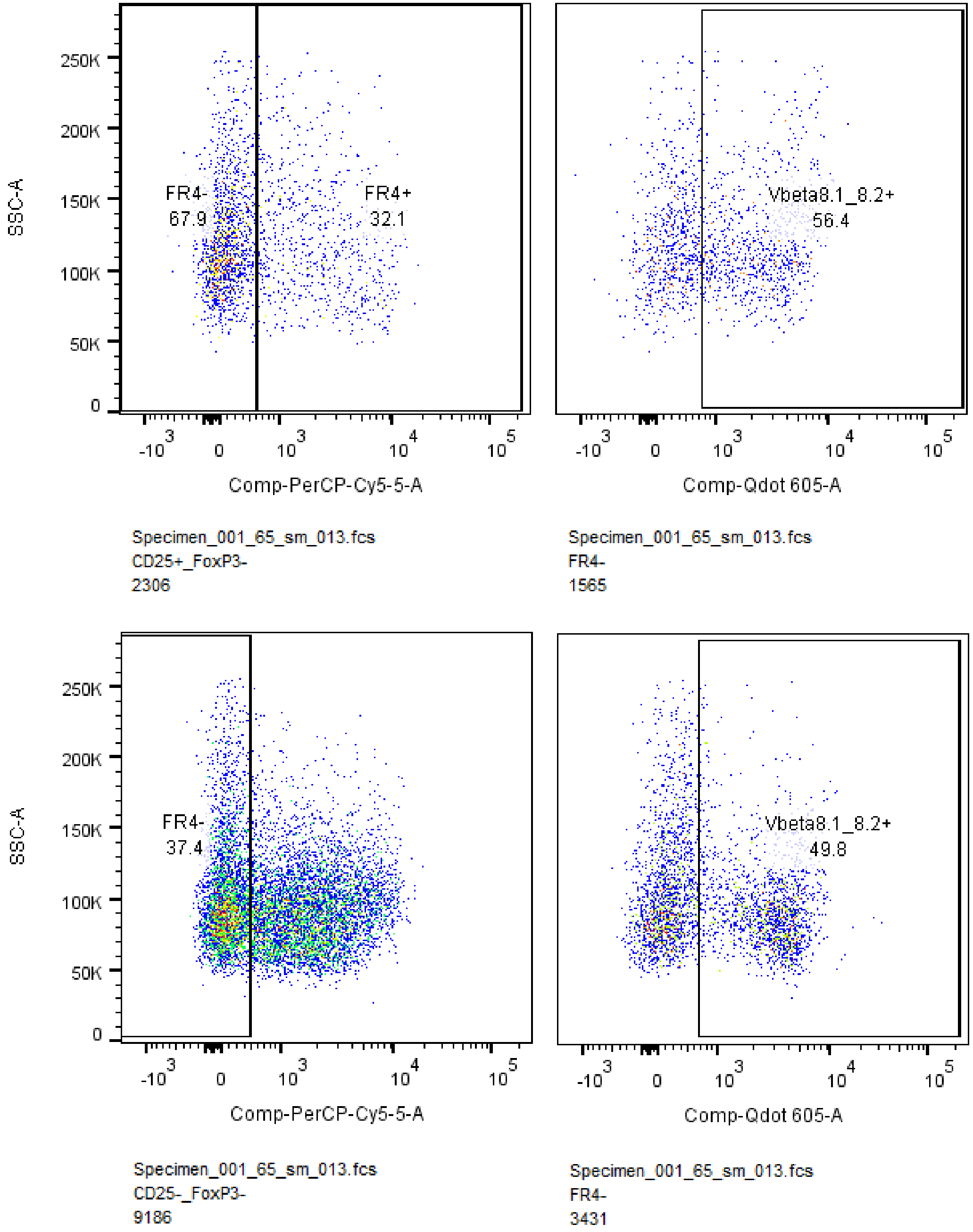


## Eye-draining lymph nodes

**Supplementary Figure 1 f.** *Population-based flow cytometry gating strategy for phenotypic characterisation of T effector cells ($T_{eff}$) in P33 dTg HEL/TCR eye-draining lymph nodes.* $T_{eff}$ cells were defined as CD4+ CD25-/+ FoxP3- FR4-. Gating for Vβ8.1/8.2+ $T_{eff}$ cells is also provided. Gate percentages refer to the cell number contained in the respective parent gate, as provided in each plot legend. This gating strategy was applied on the data presented in Figure 6.

# Supplementary Figure 2

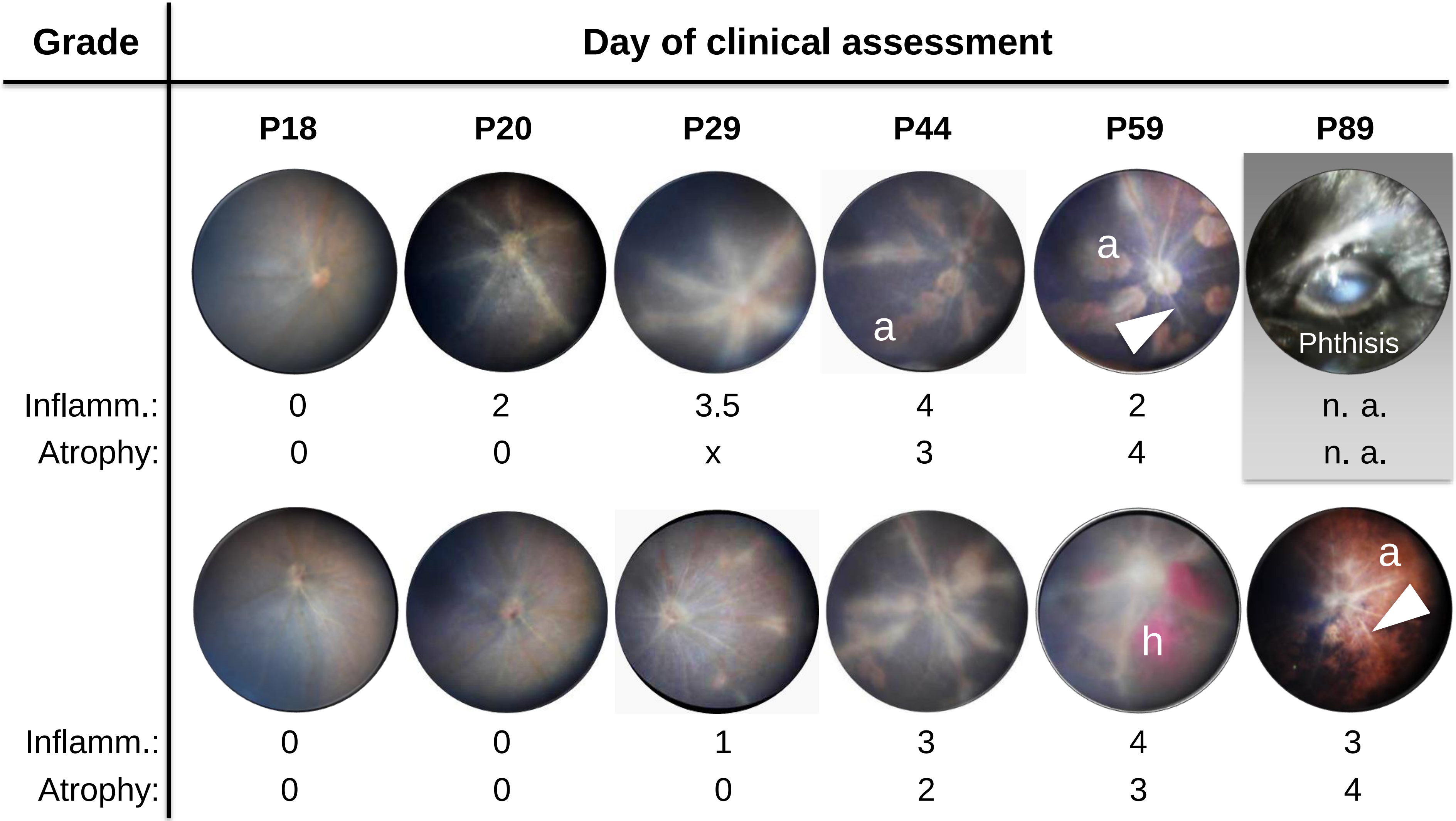


**Supplementary Figure 2.** *Clinical fundus images of progressive ocular inflammation (EAU) in dTg HEL/TCR mice imaged using an otoscope light-based system (fundoscopy)*. The upper panel images are representative of the earliest time of onset of EAU (post-partum day P20), the lower panel of a later onset (P29). Variability in disease onset and severity is shown in Figure 1A. Disease developed as focal patches of vasculitis (see lower panel, P29), leading to whole vessel involvement, haemorrhage (lower panel, P59, “h”). Burnt out disease appeared as ghost vessels (arrow head, upper panel, P59; lower panel P89) which by P89 appeared as “pipe-stem” (lower panel, P89). Large patches of paravenous retinal atrophy spread to occupy extensive retinal areas (upper panel P44, P59, “a”; lower panel P89). Some mice (~30 %) developed phthisis bulbi (upper panel, P89, grey box). Obscured retinal detail in some images is due to the presence of “vitreous haze” (*i.e.* inflammatory cell infiltration and haemorrhage into the vitreous gel, see histology in Figure 1B).

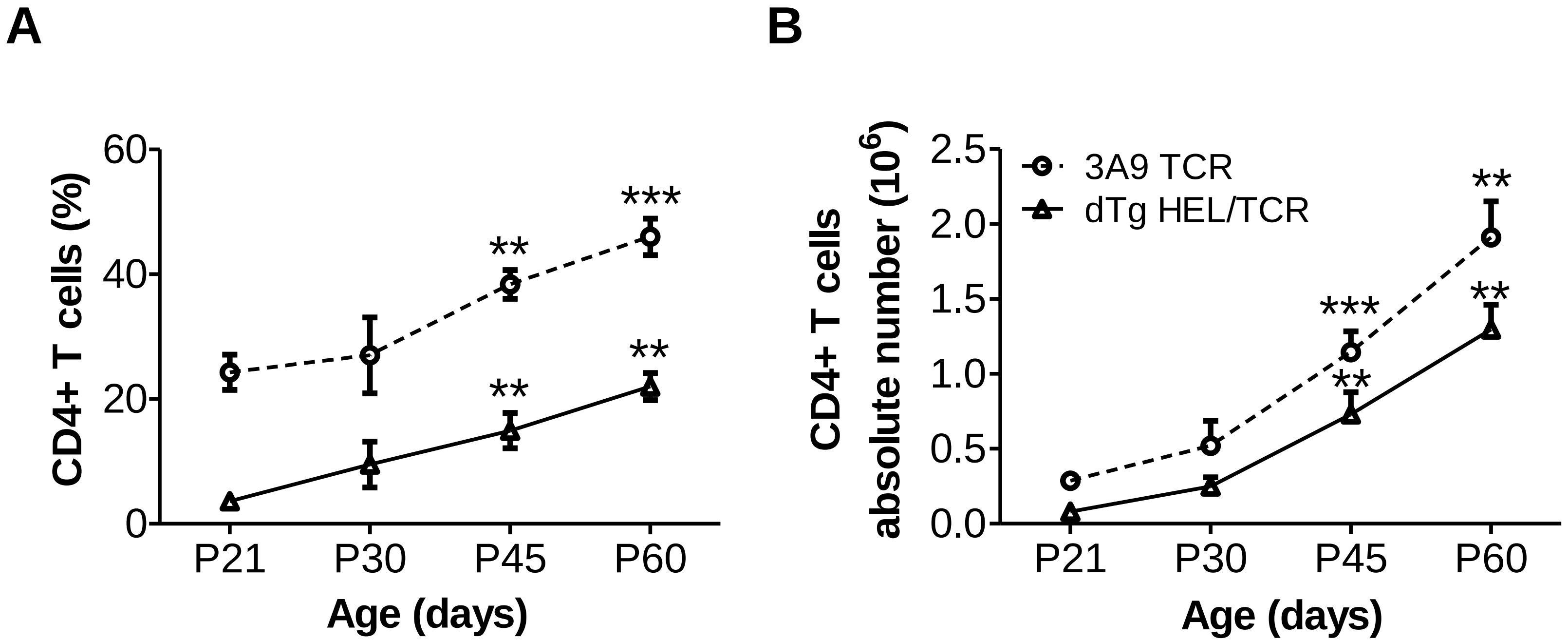


**Supplementary Figure 3.** *dTg HEL/TCR mice retain CD4+ T cell lymphopenia throughout adulthood.* CD4+ T cells found in the eye-draining lymph nodes of 3A9 TCR and dTg HEL/TCR mice of different age groups (post-partum day P21, 30, 45, and 60) were quantified using flow cytometry, and are expressed as **(A)** mean percentages, or **(B)** absolute numbers. **$p$<0.01, ***$p$<0.001 on a 95 % level of confidence.

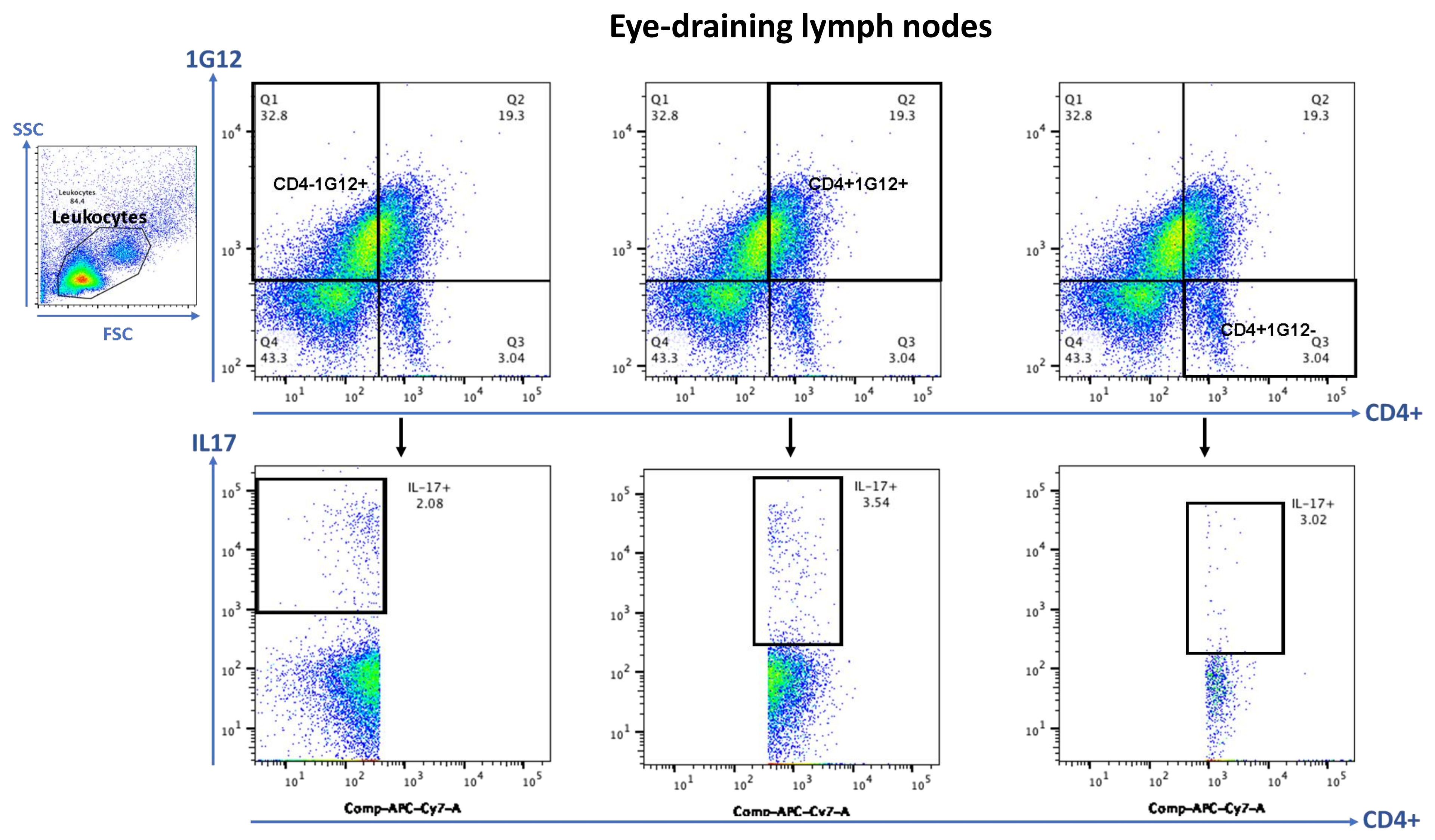


**Supplementary Figure 4 a.** *Population-based flow cytometry gating strategy for phenotypic characterisation of intracellular cytokines expression.* The figure shows intracellular IL17 expression in CD4-1G12+, CD4+1G12+, and CD4+1G12- eye-draining lymph node cells of dTg HEL/TCR mice aged P45. This strategy applies to all data provided in in Figure 2B. Gating was based on respective FMO controls (signal for each fluorochrome < 1 % of parent; APC-Cy7 for CD4, FITC for 1G12, and PE-CF594 for IL17) with respective gate percentages given.

## Supplementary Figure 4 b

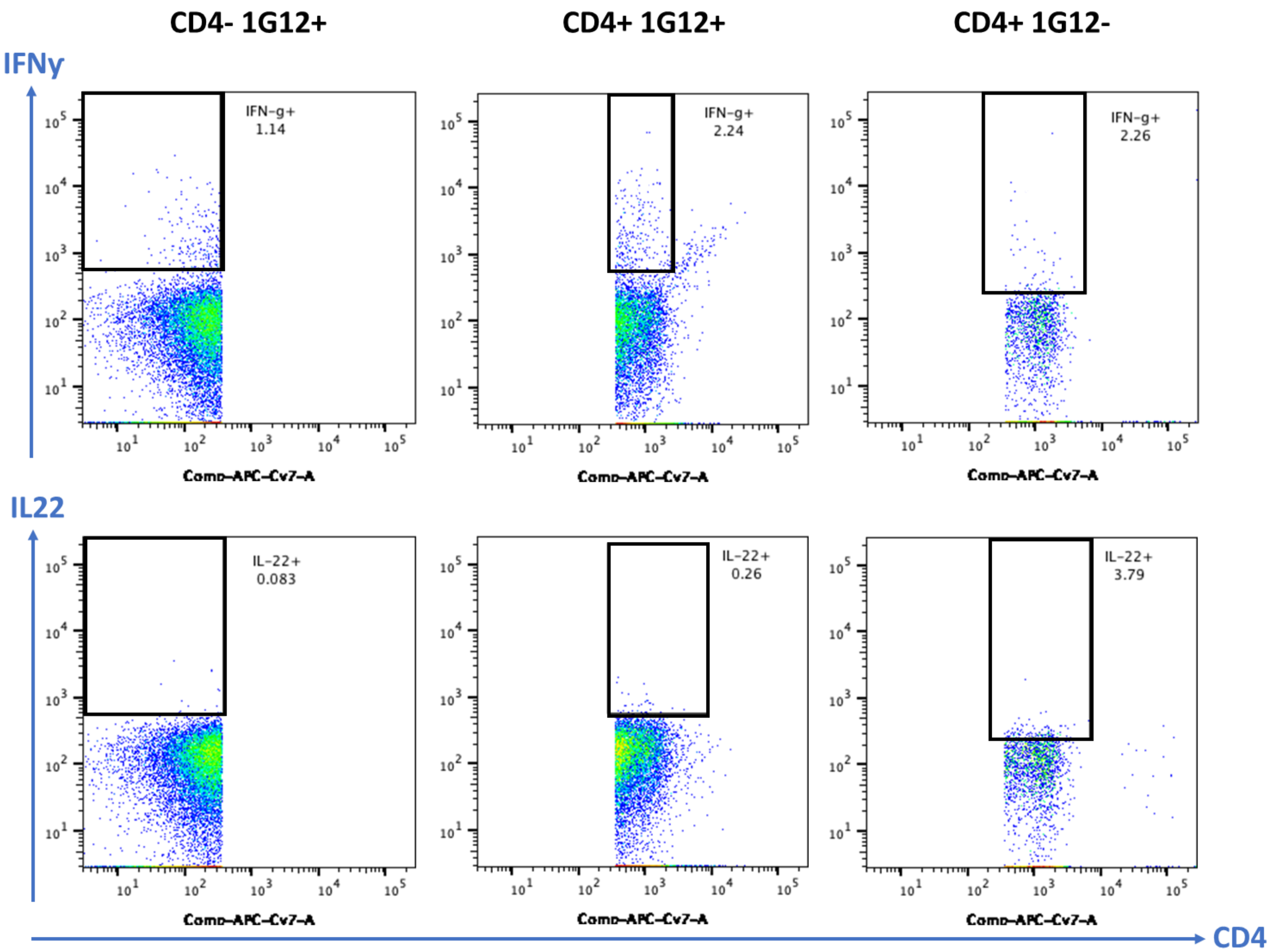


**Supplementary Figure 4 b.** *Population-based flow cytometry gating strategy for phenotypic characterisation of intracellular cytokines expression.* The figure shows intracellular IFNγ and IL22 expression in CD4-1G12+, CD4+1G12+, and CD4+1G12- eye-draining lymph node cells of dTg HEL/TCR mice aged P45. This strategy applies to all data provided in in Figure 2B. Gating was based on respective FMO controls (signal for each fluorochrome < 1 % of parent; APC-Cy7 for CD4, FITC for 1G12, APC for IFNγ, and PE for IL22) with respective gate percentages given. Initial gating was performed as shown in Supplementary Figure 4 a (upper panel).

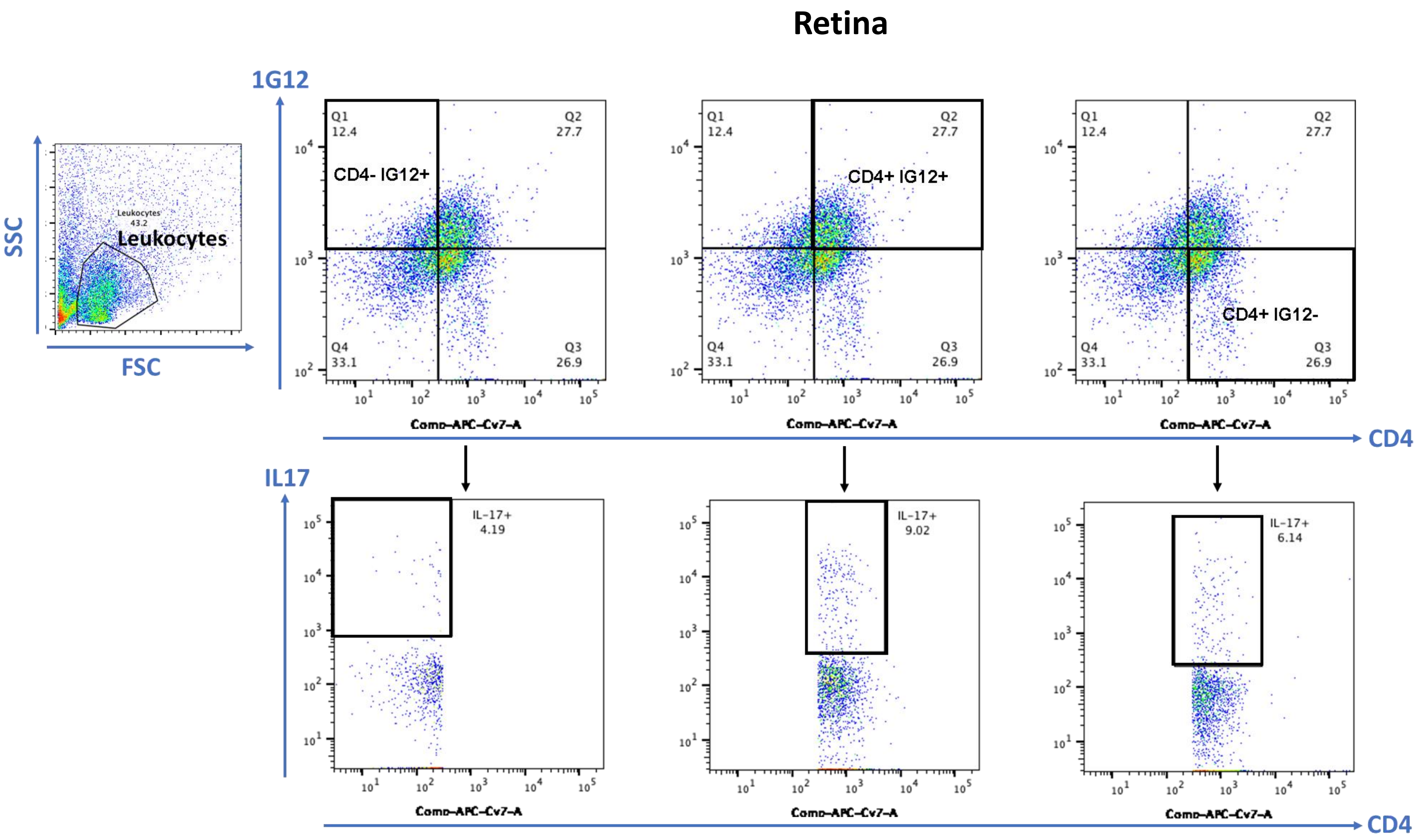


**Supplementary Figure 4 c.** *Population-based flow cytometry gating strategy for phenotypic characterisation of intracellular cytokines expression.* The figure shows intracellular IL17 expression in CD4-1G12+, CD4+1G12+, and CD4+1G12- retina cells of dTg HEL/TCR mice aged P45. This strategy applies to all data provided in in Figure 2B. Gating was based on respective FMO controls (signal for each fluorochrome < 1 % of parent; APC-Cy7 for CD4, FITC for 1G12, and PE-CF594 for IL17) with respective gate percentages given.

# Supplementary Figure 4 d

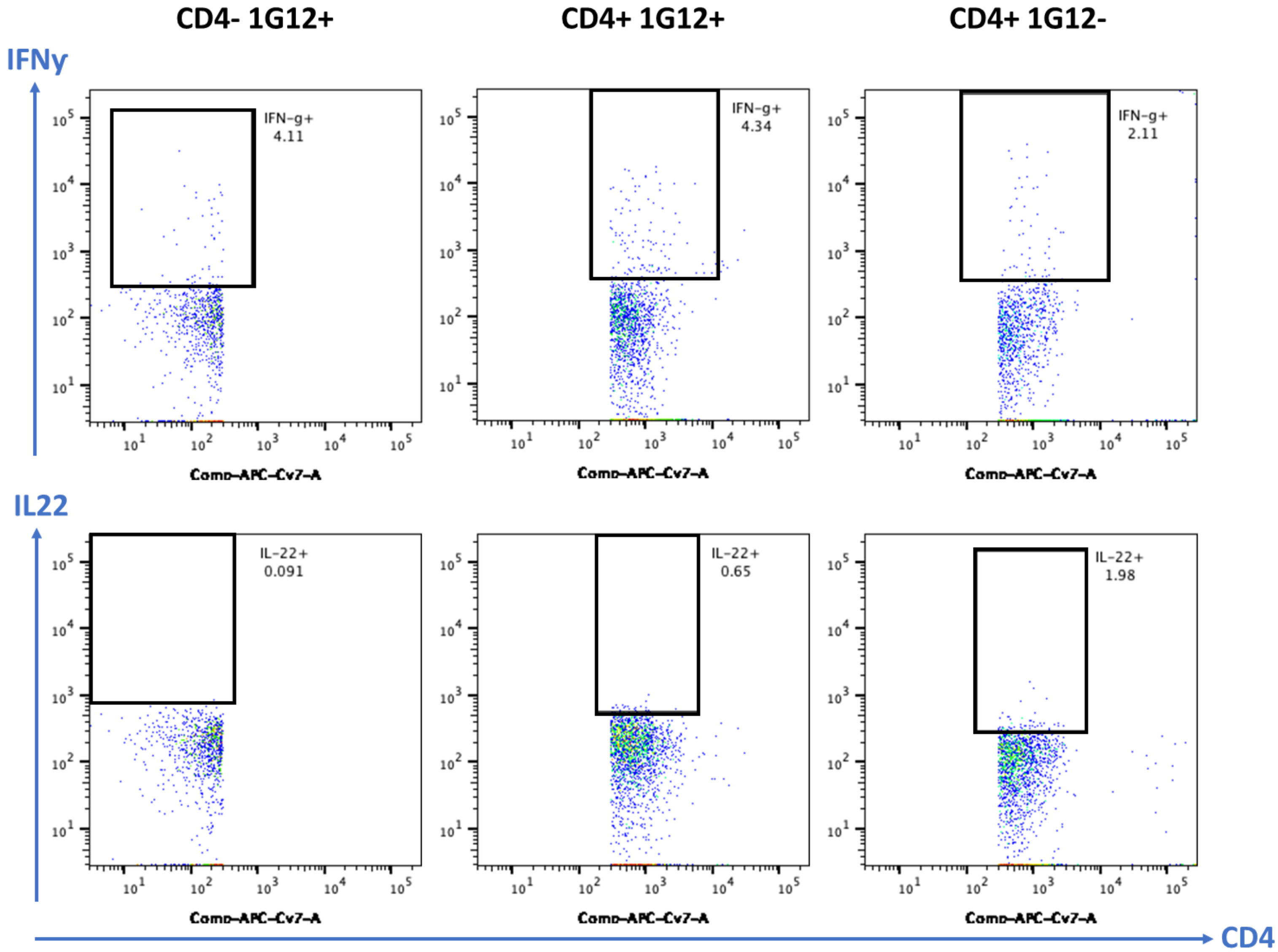


**Supplementary Figure 4 d.** *Population-based flow cytometry gating strategy for phenotypic characterisation of intracellular cytokines expression.* The figure shows intracellular IFNy and IL22 expression in CD4-1G12+, CD4+1G12+, and CD4+1G12- retina cells of dTg HEL/TCR mice aged P45. This strategy applies to all data provided in in Figure 2B. Gating was based on respective FMO controls (signal for each fluorochrome < 1 % of parent; APC-Cy7 for CD4, FITC for 1G12, APC for IFNy, and PE for IL22) with respective gate percentages given. Initial gating was performed as shown in Supplementary Figure 4 c (upper panel).

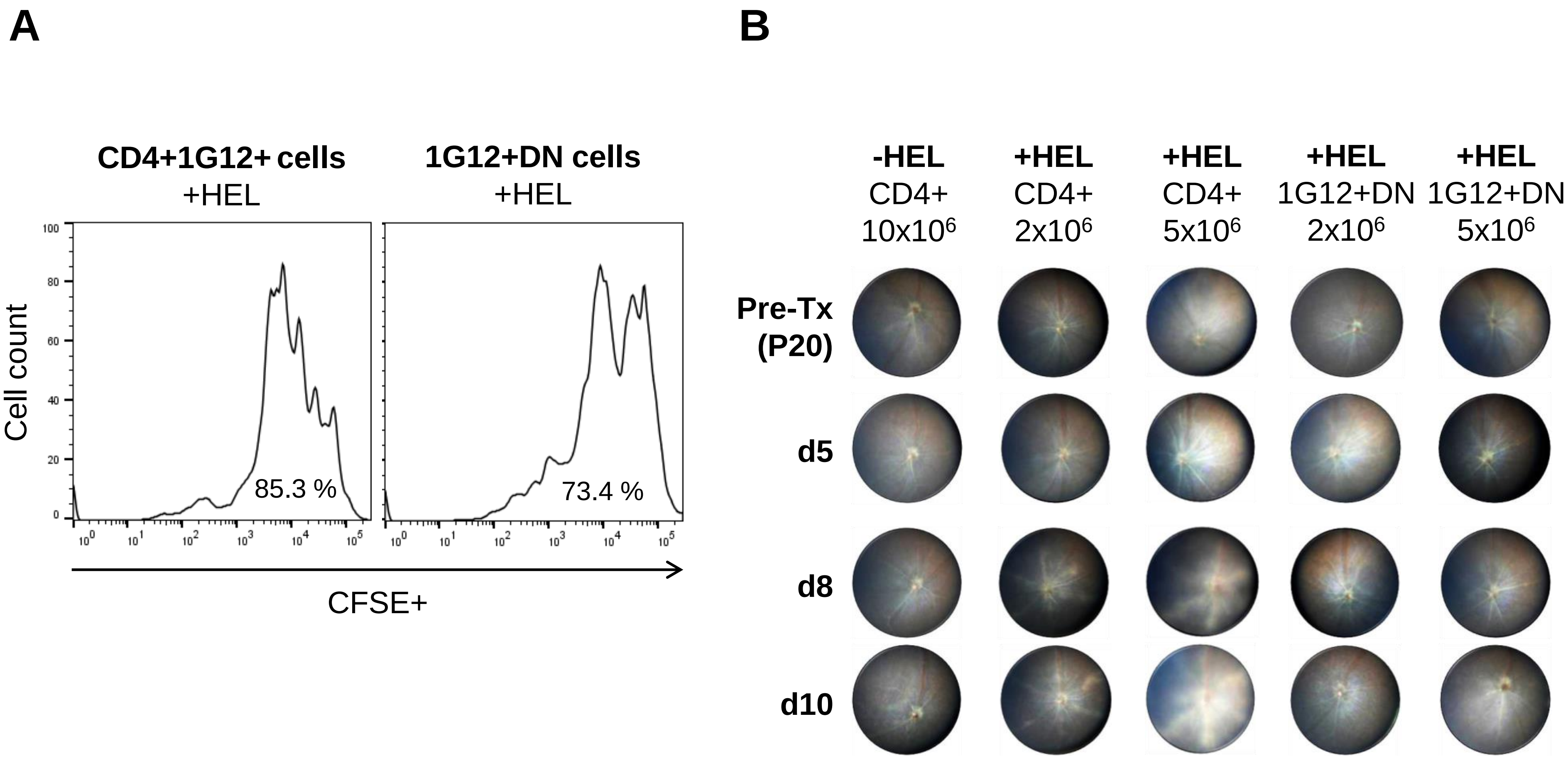


**Supplementary Figure 5.** *Cell proliferation and disease induction by different T cell subsets in IRBP:HEL sTg mice.* **(A)** Both subsets of T cells (CD4+1G12+ and 1G12+ double negative, DN) proliferated *in vitro* in response to HEL protein (1 µM over 72 hours), DN cells less so. Proliferation was assessed by flow cytometry (CFSE dilution assay) following incubation with HEL protein. **(B)** Fundus images of uveitis (EAU) in IRBP:HEL sTg mice after adoptive transfer (P21) of different cell types activated or not with HEL protein. In contrast to HEL-activated CD4+ T cells, HEL-activated antigen-specific 1G12+DN cells failed to induce disease at any time up to 10 days post-delivery.

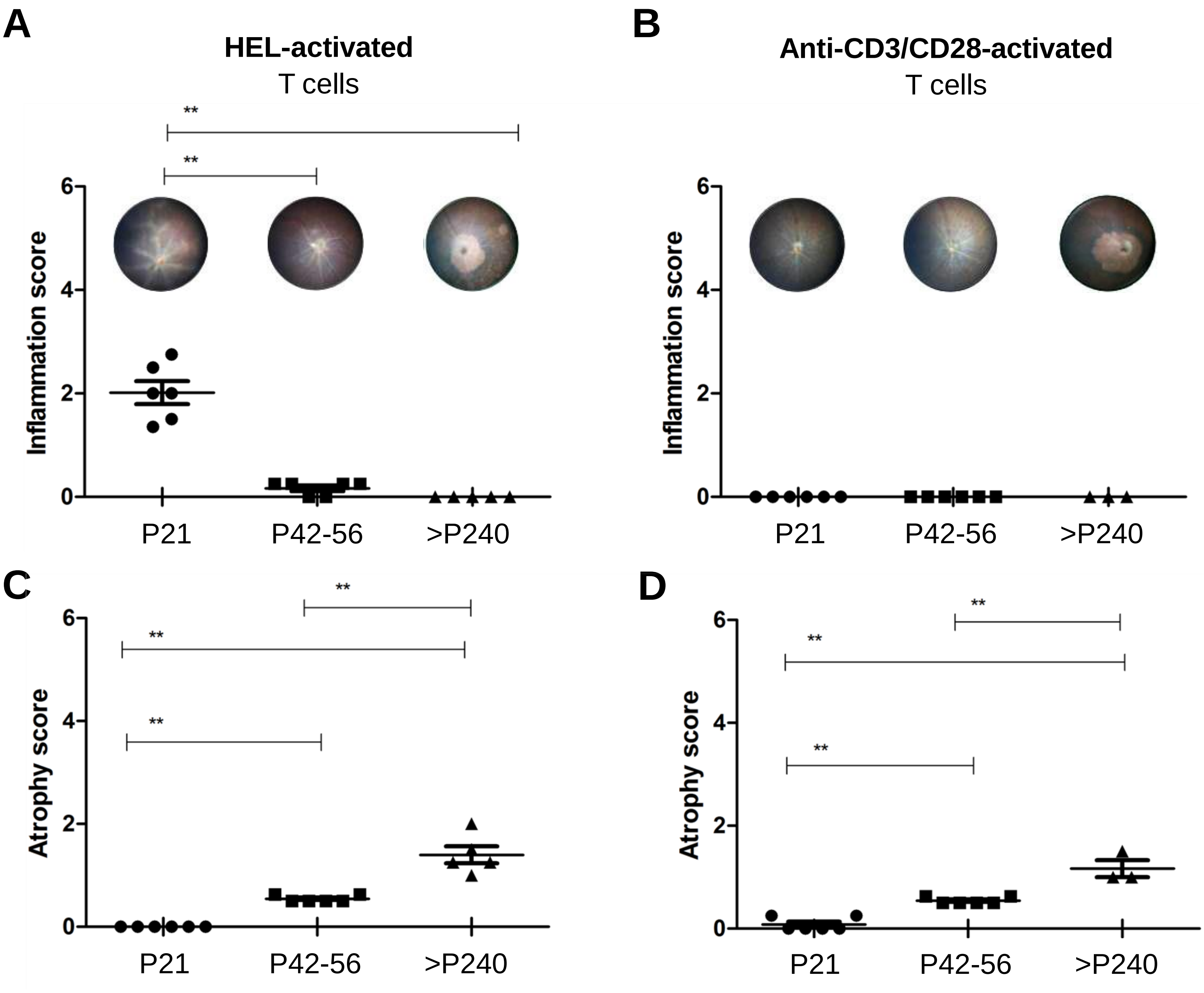


**Supplementary Figure 6.** *Adoptive transfer (Tx) of HEL-activated 3A9 T cells into IRBP:HEL sTg mice of different ages induces markedly different level of disease eight days after Tx.* sTg mice of different age groups (days post-partum) were injected once with 3A9 unfractionated lymphocyte populations ($4.5x10^6$ cells/mouse) that had been either specifically activated with **(A)** HEL protein (1 µM over 72 hours), or non-specifically with **(B)** anti-CD3/CD28 antibody (+ 200 pg/ml rIL2). For each age group, retinal inflammation **(A, B)** and atrophy **(C, D)** were clinically assessed by fundoscopy. Statistics were completed using one-way ANOVA and Tukey *post-hoc* test with *p<0.05.

# Supplementary Table 3

| Age (day post - partum) | CD4+ T cells absolute number ($\times 10^5$) in the eye-draining LN | | |
|---|---|---|---|
| | 3A9 TCR | dTg HEL/TCR | *p*-value |
| **P21** | 2.86 ± 0.4 | 0.80 ± 0.22 | 0.0001 |
| **P30** | 5.18 ± 1.6 | 2.50 ± 0.6 | 0.0230 |
| **P45** | 11.45 ± 1.4 | 7.29 ±1.47 | 0.0064 |
| **P60** | 19.11 ± 2.4 | 12.96 ±1.65 | 0.0055 |

**Supplementary Table 3.** *Flow cytometric quantification of CD4+ T cells found in the eye-draining lymph nodes (LN) of 3A9 TCR and dTg HEL/TCR mice of different age groups.* Means ± SD are presented. Significances were assessed using an unpaired *t*-test on a 95 % level of confidence.

# Supplementary Table 4

**A**

| Age (day post-partum) | $T_{reg}$ cells, absolute number (×$10^4$) in the eye-draining LN | | |
|---|---|---|---|
| | **3A9 TCR** | **dTg HEL/TCR** | ***p*-value** |
| **P21** | 4.46 ± 2.12 | 0.22 ± 0.1 | <0.0001 |
| **P30** | 12.47 ± 2.5 | 5.37 ± 2.2 | 0.0008 |
| **P45** | 16.82 ± 11.6 | 7.65 ±1.5 | n. s. |
| **P60** | 16.55 ± 6.1 | 10.34 ±3.4 | n. s. |

**B**

| Age (day post-partum) | $T_{reg}$ cells, absolute number (per retina) in the retinas | | |
|---|---|---|---|
| | **3A9 TCR** | **dTg HEL/TCR** | ***p*-value** |
| **P21** | 7.83 ± 4.6 | 738.57 ± 502.3 | <0.0001 |
| **P30** | 27.67 ± 16.8 | 3387.14 ± 1781.2 | <0.0001 |
| **P45** | 36.17 ± 33.5 | 4322.83 ± 1917.1 | <0.0001 |
| **P60** | 40.00 ± 7.2 | 4355.67 ± 1743.9 | <0.0001 |

**Supplementary Table 4.** *Flow cytometric quantification of FoxP3+CD25$^{hi}$ T regulatory cells ($T_{reg}$) found in* ***(A)*** *the eye-draining lymph nodes (LN) and* ***(B)*** *the retinas of 3A9 TCR, and dTg HEL/TCR mice of different age groups (post-partum day, P).* Means ± SD are presented. Significances were assessed using an unpaired *t*-test on a 95 % level of confidence ("n. s." describes non-significant results).

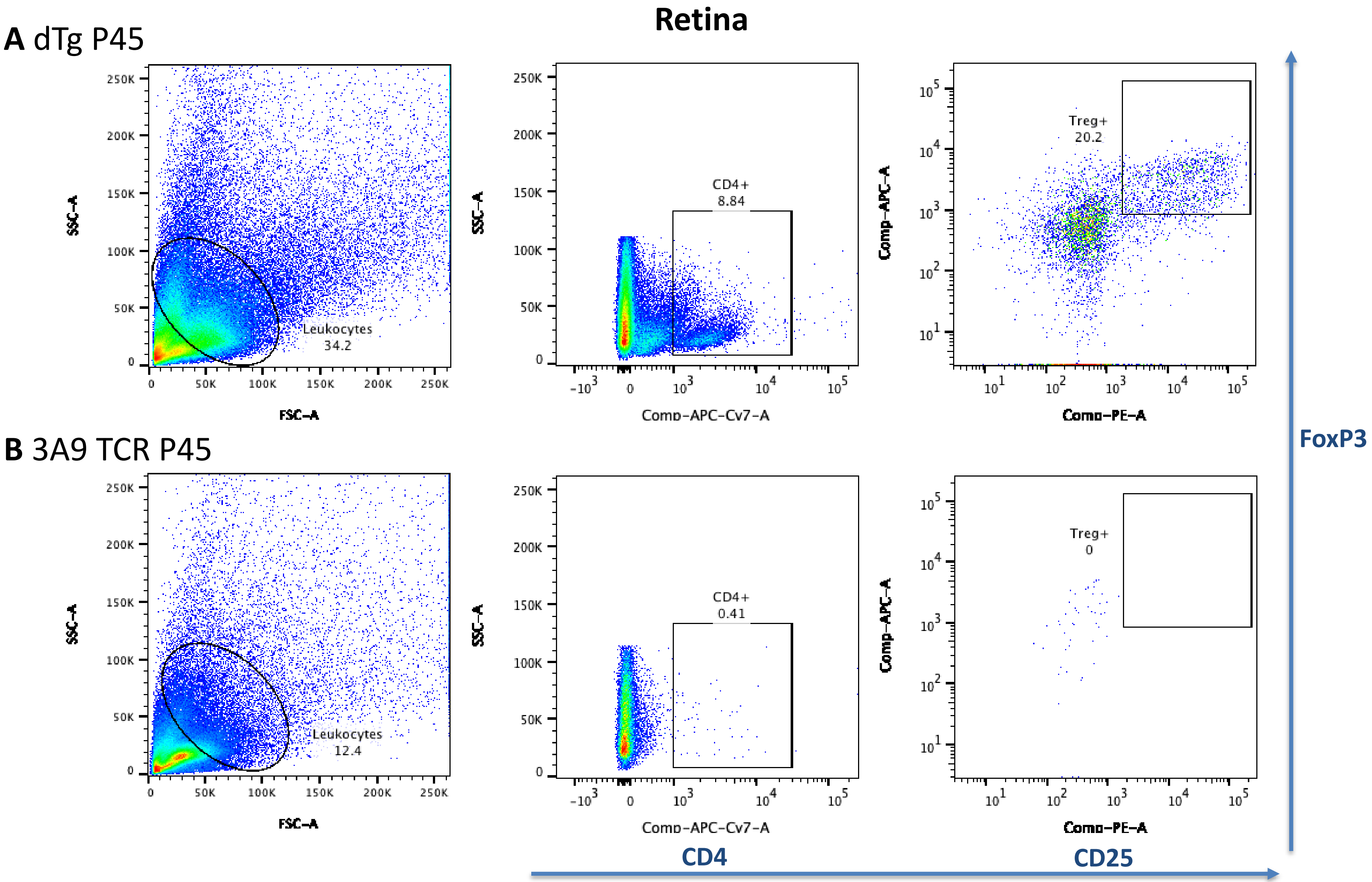


**Supplementary Figure 7 a.** *Population-based flow cytometry gating strategy for phenotypic characterisation of CD4+CD25+FoxP3+ $T_{reg}$ cells in retinas of A) P45 dTg HEL/TCR and B) sTg 3A9 TCR mice.* This strategy applies to all data provided in in Figure 5. Gating was based on respective FMO controls (signal for each fluorochrome < 1 % of parent) with gate percentages given.

# Supplementary Figure 7 b

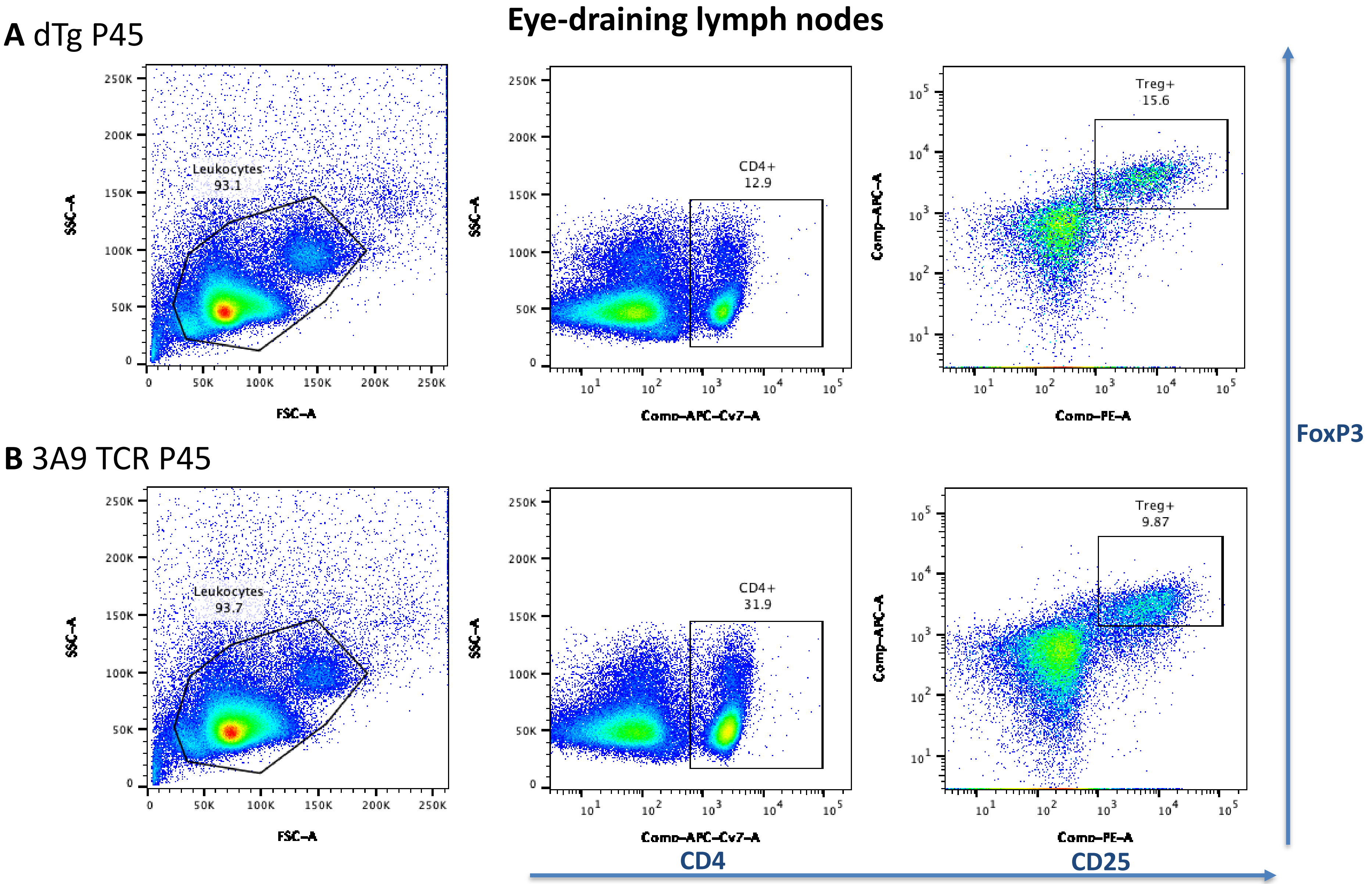


**Supplementary Figure 7 b.** *Population-based flow cytometry gating strategy for phenotypic characterisation of CD4+CD25+FoxP3+ $T_{reg}$ cells in eye-draining lymph nodes of A) P45 dTg HEL/TCR and B) sTg 3A9 TCR mice.* This strategy applies to all data provided in in Figure 5. Gating was based on respective FMO controls (signal for each fluorochrome < 1 % of parent) with gate percentages given.

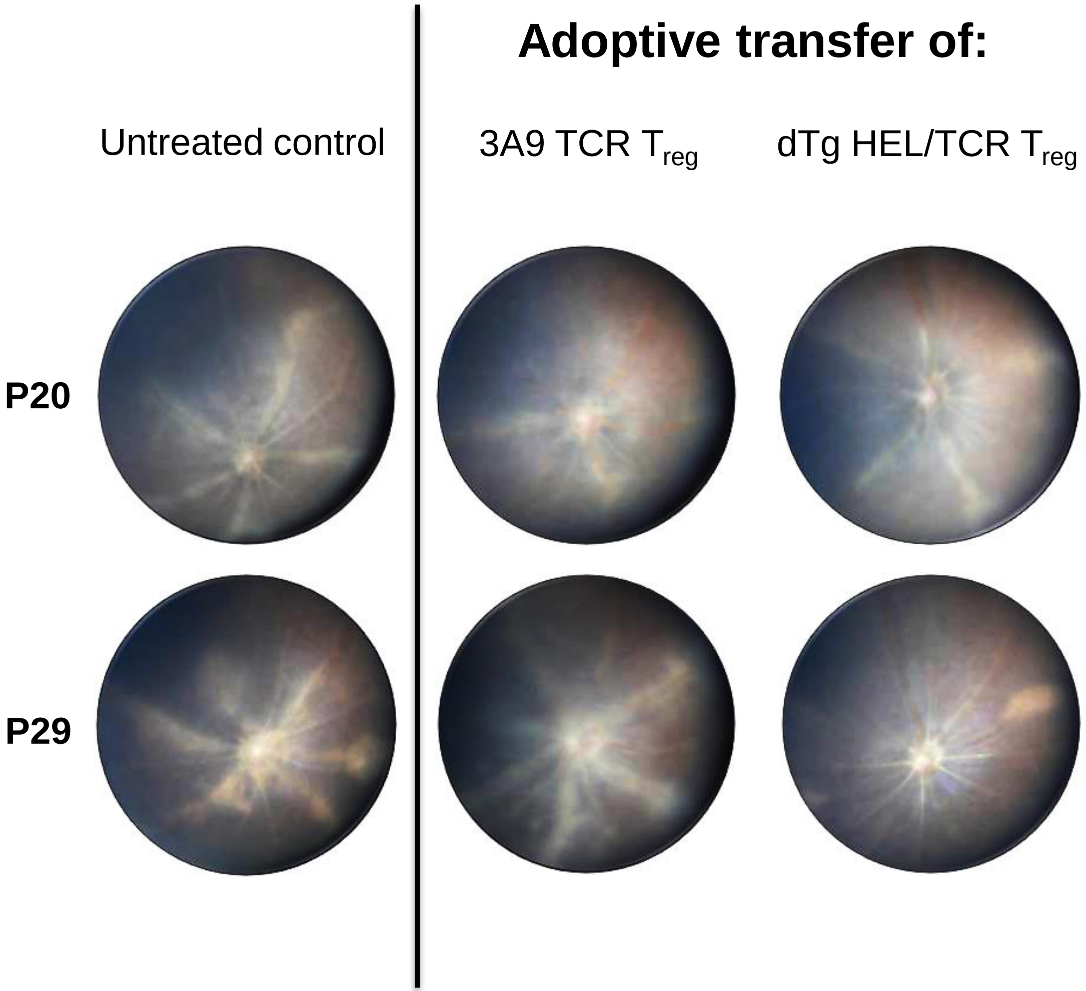


**Supplementary Figure 8.** *Adoptive transfer (Tx) of FoxP3+ T regulatory cells ($T_{reg}$) arrests EAU progression in dTg HEL/TCR mice.* $T_{reg}$ were isolated from 3A9 TCR mice aged P50-70 (naïve $T_{reg}$), and dTg mice (antigen-experienced $T_{reg}$), and adoptively transferred to post-partum day P21 dTg mice ($1x10^6$/mouse i. v.). Fundus images were taken on the day before Tx (P20) and 9 days later (P29).
